# Comprehensive antibody and cytokine profiling in hospitalized COVID-19 patients in relation to clinical outcomes in a large Belgian cohort

**DOI:** 10.1038/s41598-023-46421-4

**Published:** 2023-11-07

**Authors:** Pieter Ruytinx, Patrick Vandormael, Judith Fraussen, Zoë Pieters, Stef Thonissen, Niels Hellings, Piet Stinissen, Ina Callebaut, Joris Penders, Karolien Vanhove, Davy Kieffer, Jean-Luc Rummens, Tom Valkenborgh, Peter Messiaen, Björn Stessel, Dieter Mesotten, Veerle Somers

**Affiliations:** 1 Department of Immunology and Infection, UHasselt, Biomedical Research Institute, Martelarenlaan 42, 3500 Hasselt, Belgium; 2 Data Science Institute, UHasselt, I-BioStat, Martelarenlaan 42, 3500 Hasselt, Belgium; 3 Faculty of Medicine and Life Sciences, UHasselt, Martelarenlaan 42, 3500 Hasselt, Belgium; 4 Faculty of Medicine and Life Sciences, UHasselt, LCRC, Martelarenlaan 42, 3500 Hasselt, Belgium; 5 Department of Intensive Care and Anesthesiology, Jessa Hospital, Hasselt, Belgium; 6 Department of Laboratory Medicine, Ziekenhuis Oost-Limburg, Genk, Belgium; 7 Department of Respiratory Medicine, AZ Vesalius Hospital, Hazelereik 51, 3700 Tongeren, Belgium; 8 Department of Clinical Biology, Sint-Trudo Hospital, Diestersteenweg 100, 3800 Sint-Truiden, Belgium; 9 Department of Laboratory Medicine, Jessa Hospital, 3500 Hasselt, Belgium; 10 UHasselt, University Biobank Limburg (UBiLim), Jessa Hospital, 3500 Hasselt, Belgium; 11 Department of Anesthesiology and Intensive Care, Noorderhart Pelt, Belgium; 12 Department of Infectious Diseases and Immunity, Jessa Hospital, 3500 Hasselt, Belgium; 13 Department of Anesthesiology, Ziekenhuis Oost-Limburg, Genk, Belgium

**Keywords:** Molecular medicine, Viral infection

## Abstract

The immune response in patients with Coronavirus Disease 2019 (COVID-19) is highly variable and is linked to disease severity and mortality. However, antibody and cytokine responses in the early disease stage and their association with disease course and outcome are still not completely understood. In this large, multi-centre cohort study, blood samples of 434 Belgian COVID-19 hospitalized patients with different disease severities (ranging from asymptomatic/mild to critically ill) from the first wave of the COVID-19 pandemic were obtained. Baseline antibody and cytokine responses were characterized and associations with several clinical outcome parameters were determined. Anti-spike immunoglobulin (Ig)G and IgM levels were elevated in patients with a more severe disease course. This increased baseline antibody response however was associated with decreased odds for hospital mortality. Levels of the pro-inflammatory cytokines IL-6, IP-10 and IL-8, the anti-inflammatory cytokine IL-10 and the antiviral cytokines IFN-α, IFN-β and IFN-λ1 were increased with disease severity. Remarkably, we found significantly lower levels of IFN-λ2,3 in critically ill patients compared to patients of the moderate and severe disease category. Finally, levels of IL-8, IL-6, IP-10, IL-10, IFN-α, IFN-β, IFN-γ and IFN-λ1 at baseline were positively associated with mortality, whereas higher IFN-λ2,3 levels were negatively associated with mortality.

## Introduction

Since its emergence at the end of 2019, infection with severe acute respiratory syndrome coronavirus 2 (SARS-CoV-2) leading to Coronavirus Disease 2019 (COVID-19) has been a major threat to the health of people around the world. As of April 2022, SARS-CoV-2 has officially infected more than 500 million people worldwide, resulting in 6 million registered deaths^1^.

The disease course of COVID-19 can vary widely from asymptomatic or mild infection to moderate, severe and critical cases with severe pneumonia, sometimes evolving into acute respiratory distress syndrome (ARDS) due to excessive inflammation and lymphocytopenia, with a considerable risk of fatality^2^. Several risk factors have been associated with an increased risk for mortality, including male sex, advanced age, and comorbidities such as diabetes, obesity and asthma^3, 4^. In addition, the heterogeneity in clinical outcome is influenced by the host immune response to SARS-CoV-2 infection.

Most individuals infected with SARS-CoV-2 produce immunoglobulin (Ig)M, IgG, and IgA antibodies against the viral spike protein (S) and nucleocapsid protein (N) within 1 to 2 weeks after symptom onset, which remain elevated following initial viral clearance^5^. The S protein contains the receptor binding domain (RBD), which mediates binding to the human Angiotensin-converting enzyme 2 (ACE2) receptor identified as the receptor for the SARS-COV-2 viral entry^6^.

The currently available evidence suggests that increased antibody levels are correlated with increased clinical disease severity^7, 8^. On the other hand, anti-SARS-CoV-2 antibody titers have been shown to be strongly correlated with in vitro virus neutralization^9, 10^. Despite the well-established relationship between antibody response to SARS-CoV-2 and severity, the association with mortality is still unclear. Higher serum IgM levels have been correlated with a higher mortality rate in severe/critical COVID-19 patients^11^, whereas other studies showed that lower antibody levels are linked with increased mortality^12, 13^.

Besides the generation of an adaptive immune response, SARS-CoV-2 infection triggers an innate immune response characterized by the production of cytokines. In severe COVID-19-associated ARDS, it is known that a “cytokine storm”, an aggressive inflammatory response with the high-level release of cytokines, is an important driver of disease progression and death^14^.

Early in the pandemic, it already became clear that circulating levels of numerous inflammatory cytokines, such as interleukin (IL)-1β, IL-8, IL-6, IL-10, tumor necrosis factor (TNF)-α, interferon (IFN)-γ-inducible protein 10 (IP-10), granulocyte macrophage-colony stimulating factor (GM-CSF), and monocyte chemoattractant protein-1 (MCP-1) were increased in patients with SARS-CoV-2 infection^15^. Among the pro-inflammatory cytokines, IL-6 is considered as a key mediator of the cytokine response upon COVID-19 infection: IL-6 levels have been found to be an important predictor of COVID-19 severity and play a pivotal role in the high mortality rate^16, 17^. In addition to IL-6, also IL-1β, IL-2, TNF-α, IP-10, GM-CSF, MCP-1 and the anti-inflammatory cytokine IL-10 have been found to correlate with disease severity^18^.

A class of cytokines which has received extra attention during the COVID-19 pandemic are the interferons (IFNs), which are essential antiviral cytokines, serving as the first line of immune defence against viral infections. However, to date, conflicting evidence on the interferon response after SARS-CoV-2 infection has been reported. Recent studies showed that patients with severe COVID-19 have defective IFN responses^19, 20^, however other studies showed an increased and prolonged production of IFNs in patients infected with SARS-CoV-2, which was, in turn, correlated with negative clinical outcomes^21, 22^.

In order to address these questions, a large multi-centre cohort study was set-up to collect blood samples from hospitalized COVID-19 patients during the first wave of the COVID-19 pandemic to thoroughly profile the immune response by analysing SARS-CoV-2-specific antibodies and a panel of cytokines at baseline. Next, the association between these early immune responses and clinical outcome and laboratory parameters was determined.

## Results

### Patient demographics, clinical characteristics and clinical outcomes

A retrospective cross-sectional study was performed using blood samples from 434 COVID-19 patients hospitalized during the first COVID-19 wave between April and June 2020. A systematic COVID-19 PCR screening was performed at hospital admission regardless of the reason for hospitalization. Blood samples were collected at baseline, which is defined as within 4 days after a positive PCR for antibody detection and within 8 days after a positive PCR for cytokine measurement. Demographics, disease characteristics and comorbidities at baseline are reported in Table 1. COVID-19 patients were categorized according to WHO classification criteria (WHO clinical management of COVID-19 interim guidance issued May 27 2020, Table 2), into mild/asymptomatic (n = 54), moderate (n = 196), severe (n = 99) and critically ill (n = 68) disease categories. For 17 patients, information regarding their disease category at baseline blood sampling was lacking. The median (IQR) age of the patients was 71,0 years (57,0–81,8), 245 (56.5%) were men, and the median (IQR) BMI was 27.7 (24.3–31.2) (Table 1). No significant differences in age were found between the different disease severity categories (p = 0.1877). A statistically significant difference was found for sex, with a significantly higher number of male patients in the critically ill group and moderate group compared to the patient group with an asymptomatic/mild category at baseline (p = 0.0156 and p < 0.0001 respectively). BMI was significantly different between the different disease severity categories (p = 0.0282) with a significantly higher BMI in the severe group compared to the asymptomatic/mild group (p = 0.0192). The most common comorbidities were arterial hypertension (n = 181 patients, 42.8%), diabetes (n = 81 patients, 18.8%), kidney injury (n = 74 patients, 17.2%), and malignancies (n = 53 patients, 12.2%) (Table 1). Next, the differences in laboratory parameters between disease severities were investigated, focussing only on differences between patients from the mild, moderate or severe disease category with the critically ill group. Among the laboratory parameters, we found at baseline that the acute phase reactants C-reactive protein (CRP) and ferritin levels were significantly different in critically ill patients compared to patients within the moderate (p < 0.0001 and p = 0.0074 respectively) and mild disease category (p < 0.0001 and p = 0.0003 respectively), more specifically critically ill patients displayed higher CRP and ferritin levels. Furthermore, white blood cell count was significantly different in critically ill patients compared to patients belonging to the moderate disease category (p = 0.0199), with critically ill patients displaying a higher white blood cell count. No significant differences were found for the level of the coagulation dysfunction marker D-dimer, between the different categories (p = 0.1374). The PF ratio, indicating the respiratory function was significantly different in critically ill patients compared to patients with a severe (p = 0.0025), moderate (p < 0.0001) and mild (p = 0.0017) disease at baseline, observing a lower PF ratio among critically ill patients.

**Table 1:** Demographics and baseline characteristics of hospitalized patients with COVID-19 according to disease severity category.

Characteristic (N = 434)	N	Value	Disease severity category^†^			
			Asymptomatic/mild (n = 54)	Moderate (n = 196)	Severe (n = 99)	Critically ill (n = 68)
Male	434	245 (56.5)	17/54 (31.5)	114/196 (58.2)^a^	62/99 (62.6)	43/68 (63.2)^a^
Age, median (IQR) years	434	71.0 (57.0–81.8)	66.0 (54.0–85.0)	70.0 (54.0–81.0)	73.0 (63.5–82.0)	71.0 (60.0–79.0)
BMI, median (IQR) kg/m^2^	288	27.7 (24.3–31.2)	25.9 (20.7–27.5)	27.9 (24.1–32.3)	28.4 (25.9–32.7)^a^	27.3 (23.6–29.8)
**Laboratory parameters**						
CRP, median (IQR) mg/ml	411	67.0 (28.6–129.5)	21.6 (4.4–43.3)	63.6 (30.9–114.5)	92.5 (48.3–147.7)	132.1 (65.1–218.4)^a,b^
D-dimer, median (IQR) mg/L	250	0.9 (0.5–1.7)	0.9 (0.6–2.0)	0.8 (0.5–1.4)	1.0 (0.6–1.8)	1.2 (0.7–1.8)
Ferritin, median (IQR) µg/L	146	550.3 (240.0–1200.0)	235.0 (137.1–560.5)	378.0 (196.0–940.0)	595.0 (464.0–1350.0)	874.2 (515.9–2000.0)^a,b^
WBC, median (IQR) × 10^9^/L	414	6.5 (4.8–8.5)	7.2 (5.0–9.3)	6.1 (4.6–8.0)	6.6 (4.8–8.3)	7.7 (5.7–9.6)^b^
PF ratio	170	300.5 (216.9–371.5)	371.0 (342.5–404.5)	328.1 (285.7–379.5)	269.5 (214.0–348.1)	155.0 (115.0–191.4)^a,b,c^
**Comorbidities**						
Diabetes	431	81 (18.8)	3/51 (5.9)	39/196 (19.9)	23/99 (23.2)	14/68 (20.6)
Arterial hypertension	423	181 (42.8)	13/43 (30.2)	72/196 (36.7)	57/99 (57.6)	33/68 (48.5)
Kidney injury	431	74 (17.2)	1/51 (2.0)	37/196 (18.9)	22/99 (22.2)	11/68 (16.2)
Hepatic failure	431	3 (0.7)	2/52 (3.9)	0/196 (0.0)	0/98 (0)	1/68 (1.5)
Heart failure	431	4 (0.9)	0 (0.0)	3/196 (1.5)	1/99 (1.0)	0/68 (0.0)
Chronic lung disease	430	47 (10.9)	3/51 (5.9)	21/196 (10.7)	14/98 (14.3)	7/68 (10.3)
Malignancies	434	53 (12.2)	10/54 (18.5)	21/196 (10.7)	10/99 (10.1)	9/68 (13.2)
Immunocompromised status	430	29 (6.7)	4/51 (7.8)	12/195 (6.2)	3/99 (3.0)	7/68 (10.3)

Data are n (%) unless indicated otherwise, where N is the number of available data.

The differences in patient characteristics and laboratory parameters between disease severity were investigated. The Aligned Rank Transform ANOVA was carried out for continuous parameters. For discrete patient characteristics a logistic GEE model was used. The p-values of both tests were adjusted for multiple comparisons using the Tukey-Kramer method. The significance level is taken to be 5%.

*BMI* body mass index, *CRP* C-reactive protein, *WBC* white blood cell, *PF ratio* ratio of arterial oxygen partial pressure to fractional inspired oxygen.

^†^Information on category is missing for 17 patients.

^a^vs. Asymptomatic/mild cases (p < 0.05).

^b^vs. moderate cases (p < 0.05).

^c^vs. severe cases (p < 0.05).

**Table 2:** Guideline clinical management of COVID-19 patients; living guideline according to WHO, 27 May 2020.

Category	Disease phenotype
Asymptomatic/mild disease	PCR positive, no symptoms, or symptomatic patients without evidence of viral pneumonia or hypoxia
Moderate disease	Clinical signs of pneumonia (fever, cough, dyspnea, and fast breathing) but no signs of severe pneumonia, including peripheral capillary SpO2 ≥ 90% on room air
Severe disease	Patients with clinical signs of pneumonia (fever, cough, dyspnoea, fast breathing) plus one of the following: respiratory rate > 30 breaths/min; severe respiratory distress; or SpO2 < 90% on room air
Critical disease	Patients meet any of the following criteria of ARDS, sepsis, or septic shock

*SpO2* oxygen saturation, *ARDS* acute respiratory distress syndrome.

Table 3 shows the different treatment regimens and clinical outcomes of patients who were hospitalized with COVID-19 during the study period. The median (IQR) length of hospital stay was 8 days (4.7–14.0). Intensive care unit (ICU) admission was required for 85 (19.6%) patients with a median (IQR) ICU length of stay of 7.2 days (3.0–16.1), of which 21 (24.7%) patients died at ICU. Hospital mortality occurred in 80 (18.9%) patients. Seventy-five (17.7%) and 85 (20.1%) patients died within 30 and 90 days after hospital admission, respectively.

**Table 3:** Treatments and outcomes of hospitalized patients with COVID-19.

Characteristic (N = 434)	N	Value	Disease severity category^†^			
			Asymptomatic/mild (n = 54)	Moderate (n = 196)	Severe (n = 99)	Critically ill (n = 68)
**Therapies**						
Antibacterial therapy	434	346 (79.7)	22/54 (40.7)	176/196(89.8)	79/99 (79.8)	66/68 (97.1)
Antifungal therapy	434	10 (2.3)	1/54 (1.9)	1/196 (0.5)	6/99 (6.1)	2/68 (2.9)
Anti-inflammatory therapy						
IL inhibitors	434	3 (0.7)	0/54 (0.0)	0/196 (0.0)	1/99 (1.0)	2/68 (2.9)
Corticosteroids	434	65 (15.0)	5/54 (9.3)	26/196 (13.3)	14/99 (14.1)	19/68 (27.9)
Hydroxychloroquine	434	171 (39.4)	4/54 (7.4)	73/196 (37.2)	46/99 (46.5)	47/68 (69.1)
Antiviral therapy						
Lopinavir	434	3 (0.7)	0/54 (0.0)	0/196 (0.0)	2/99 (2.0)	1/68 (1.5)
Remdesivir	434	1 (0.2)	0/54 (0.0)	0/196 (0.0)	0/99 (0.0)	1/68 (1.5)
Renal therapy^∆^	434	13 (3.0)	0/54 (0.0)	3/196 (1.5)	2/99 (2.0)	6/68 (8.8)
Vasopressor use	421	26 (6.2)	0/43 (0.0)	7/195 (3.6)	5/98 (5.1)	14/68 (20.6)
Other therapy	397	175 (44.1)	27/50 (54.0)	59/175 (33.7)	35/93 (37.6)	51/62 (82.3)
**Outcome parameters**						
30-day mortality	423	75 (17.7)	3/43 (7.0)	18/196 (9.2)	19/99 (19.2)	32/68 (47.1)
90-day mortality	423	85 (20.1)	5/43 (11.6)	23/196 (11.7)	21/99 (21.2)	33/68 (48.5)
Hospital mortality	423	80 (18.9)	4/43 (9.3)	21/196 (10.7)	20/99 (20.2)	32/68 (47.1)
Hospital length of stay, median (IQR) days	423	8 (4.7–14.0)	7.0 (2.5–13.0)	7.9 (5.0–12.9)	10.0 (5.9–12.9)	12.0 (6.0–23.5)
ICU admission ever	434	85 (19.6)	1/54 (1.9)	22/196 (11.2)	21/99 (21.2)	40/68 (58.8)
ICU length of stay⃰, median (IQR) days	84	7.2 (3.0–16.0)	4.0 (4.0–4.0)	3.8 (1.1–11.2)	6.8 (4.6–31.2)	8.5 (5.4–18.3)
ICU-mortality*	85	21 (24.7)	0/1 (0.0)	5/22 (22.7)	3/21 (14.3)	12/40 (30.0)

Data are n (%) unless indicated otherwise, where N is the number of available data.

*IL* Interleukin, *ICU* intensive care unit.

*Only for patients admitted to ICU.

^†^Information on category is missing for 17 patients.

^∆^4 patients have chronic replacement therapy.

Other therapy including low-molecular weight heparins or dialysis.

### Baseline antibody response in the Limburg COVID-19 cohort

**Figure 1:**
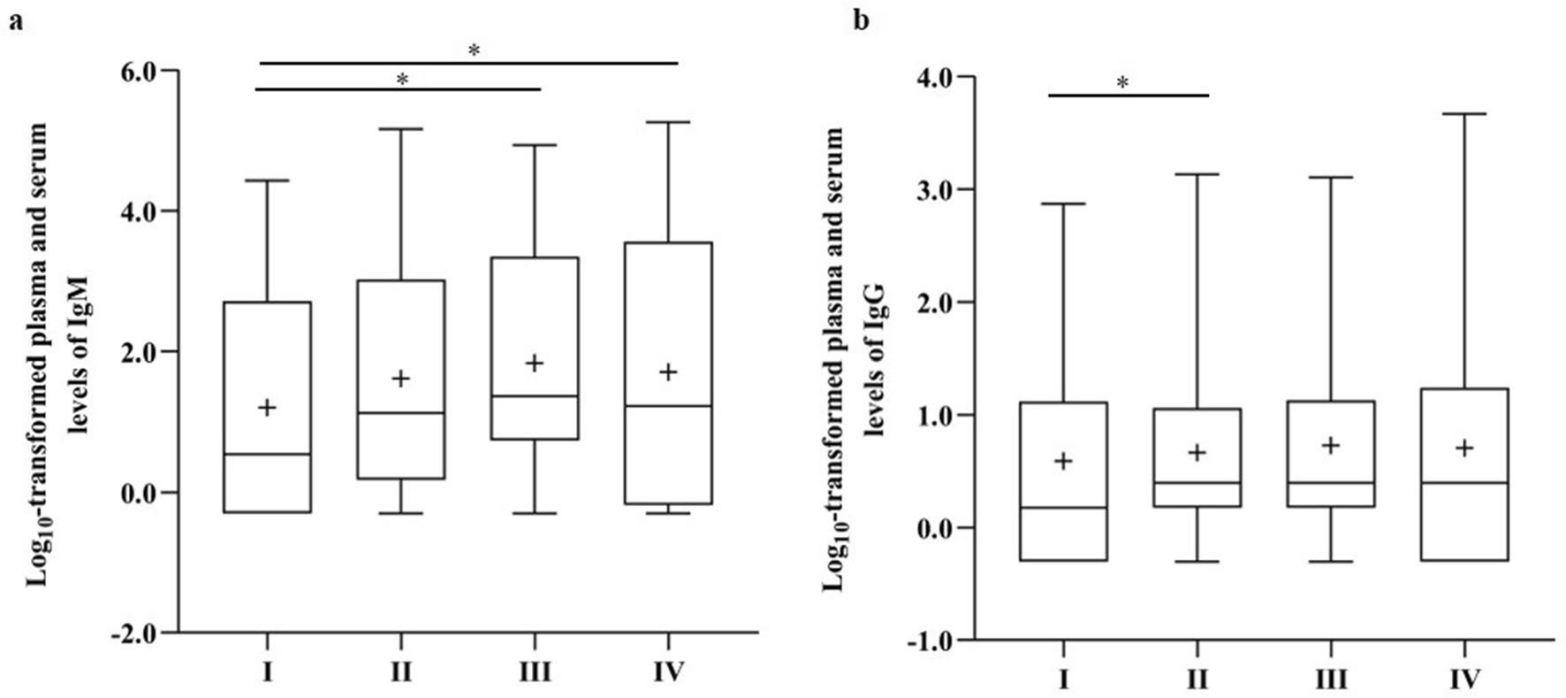
Antibody levels in COVID-19 patients according to disease severity. Comparison between log_10_-transformed plasma and serum levels of IgM (**a**) in asymptomatic/mild (I, n = 52), moderate (II, n = 180), severe (III, n = 90) and critically ill patients (IV, n = 56) and IgG levels (**b**) in asymptomatic/mild (I, n = 54), moderate (II, n = 187), severe (III, n = 97) and critically-ill patients (IV, n = 59). The box plot visualizes the following summary statistics: the middle line represents the median; the lower hinge corresponds to the first quartile (25th); the upper hinge corresponds to the third quartile (75th); upper and lower whiskers extend from the hinge respectively to the largest value and smallest value. Data are shown as median (range), the plus sign indicates the average level. Significance was calculated between the groups and p*-*values were adjusted for multiple comparisons using the Tuckey-Kramer method (*p < 0.05).

We first analysed the IgM and IgG antibody responses against the spike protein of SARS-CoV-2. At baseline, within 4 days of a positive PCR, anti-spike S1 protein antibodies of the IgM and IgG isotype were detected in 209/391 (53.5%) and 99/411 (24.1%) of patients, respectively. In a subgroup of 391 patients, for which the presence of both IgM and IgG antibodies was determined, almost all patients (181/182) who were negative for IgM isotype antibodies were also negative for IgG. 118 Patients were positive for IgM only, and almost all (91/92) patients who tested positive for IgG were also positive for IgM, reflecting the natural course of Ig response to SARS-CoV-2 spike protein (Table 4). Next, we assessed whether the log_10_-transformed antibody levels differed according to disease severity. The SARS-CoV-2 specific IgM levels of patients within the severe disease category (p < 0.0001) and critically ill patients (p = 0.0271) were significantly higher than those of patients belonging to the asymptomatic/mild subgroup (Fig. 1a). For IgG antibodies directed to the SARS-CoV-2 S1 protein, we found an overall trend towards higher IgG antibody levels in patients belonging to a more severe disease category, with a statistically significant difference between patients belonging to the moderate and the asymptomatic/mild subgroup (p = 0.0020) (Fig. 1b).

**Table 4:** Association between IgM and IgG seropositivity at baseline.

	IgM seronegative	IgM seropositive	Total
IgG seronegative	181	118	299
	60.54	39.46	
	99.45	56.46	
IgG seropositive	1	91	92
	1.09	98.91	
	0.55	43.54	
Total	182	209	391

The association between seropositivity for IgM and IgG was investigated by a frequency table. The cells contain (from top to bottom) the frequency, row percent, and column percent.

### Association between baseline antibody levels and clinical outcome parameters

**Figure 2:**
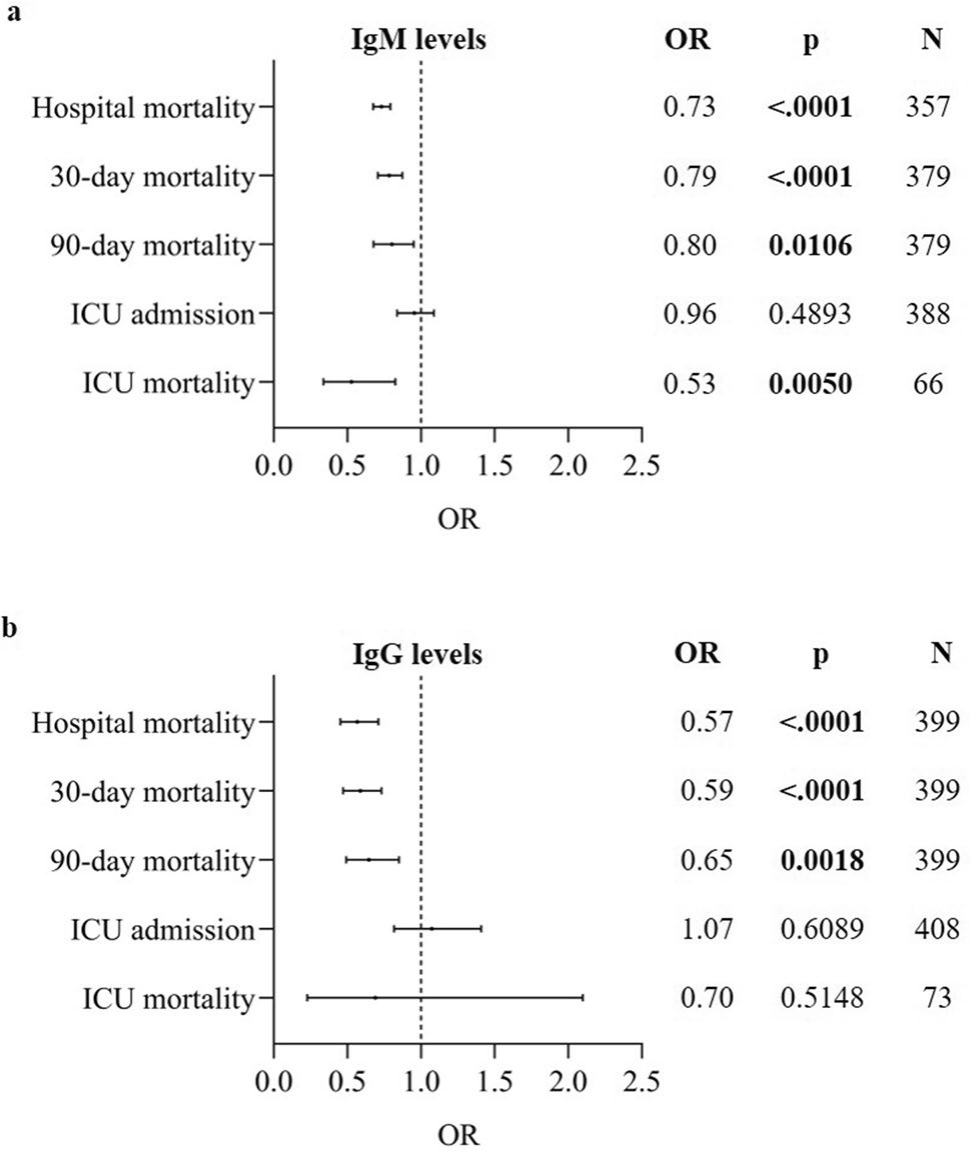
Association between baseline antibody levels and clinical outcomes. Odds ratios (ORs) are shown for the association between IgM (**a**), IgG (**b**) levels and clinical outcomes including hospital mortality, 30-day mortality, 90-day mortality, ICU admission, and ICU mortality using a forest plot. The ORs indicated in the table are calculated using the adjusted GEE model. The models were adjusted through a backward model selection procedure including sex, age, BMI, comorbidities and therapies. Lines show 95% confidence intervals. N = number of patients included in the analysis. A p-value is significant if its value is lower than the level of significance, which is set at 5% and indicated in bold.

The association between antibody levels and clinical outcome parameters was analyzed using an adjusted GEE model. The GEE model was adjusted for important covariates through a backward selection procedure including sex, age, BMI, comorbidities and therapies. The estimated OR based on the GEE model for hospital mortality was lower for patients with higher levels of IgM and IgG at baseline. For a tenfold increase in baseline IgM levels (corresponding to 1-unit increase in log_10_-transformed scale), the odds of hospital mortality decreased with 27% (OR = 0.73, 95% CI: 0.68–0.79, p < 0.0001) (Fig. 2a), while a tenfold increase in IgG levels decreased the odds of hospital mortality with 43% (OR = 0.57, 95% CI: 0.45–0.71, p < 0.0001) (Fig. 2b). In line with this observation, the adjusted model also showed lower odds for mortality within 30 and 90 days after hospitalization with increased levels of IgM or IgG (Fig. 2a,b). For ICU patients, a tenfold increase in IgM levels was associated with lower odds for ICU mortality (OR = 0.53, 95% CI 0.34–0.83, p = 0.005) (Fig. 2a). No significant associations were found between IgM and IgG antibody levels and the odds of being admitted to ICU, and between IgG levels and the odds of ICU mortality (Fig. 2a,b).

### Association between baseline antibody levels and laboratory parameters

**Figure 3:**
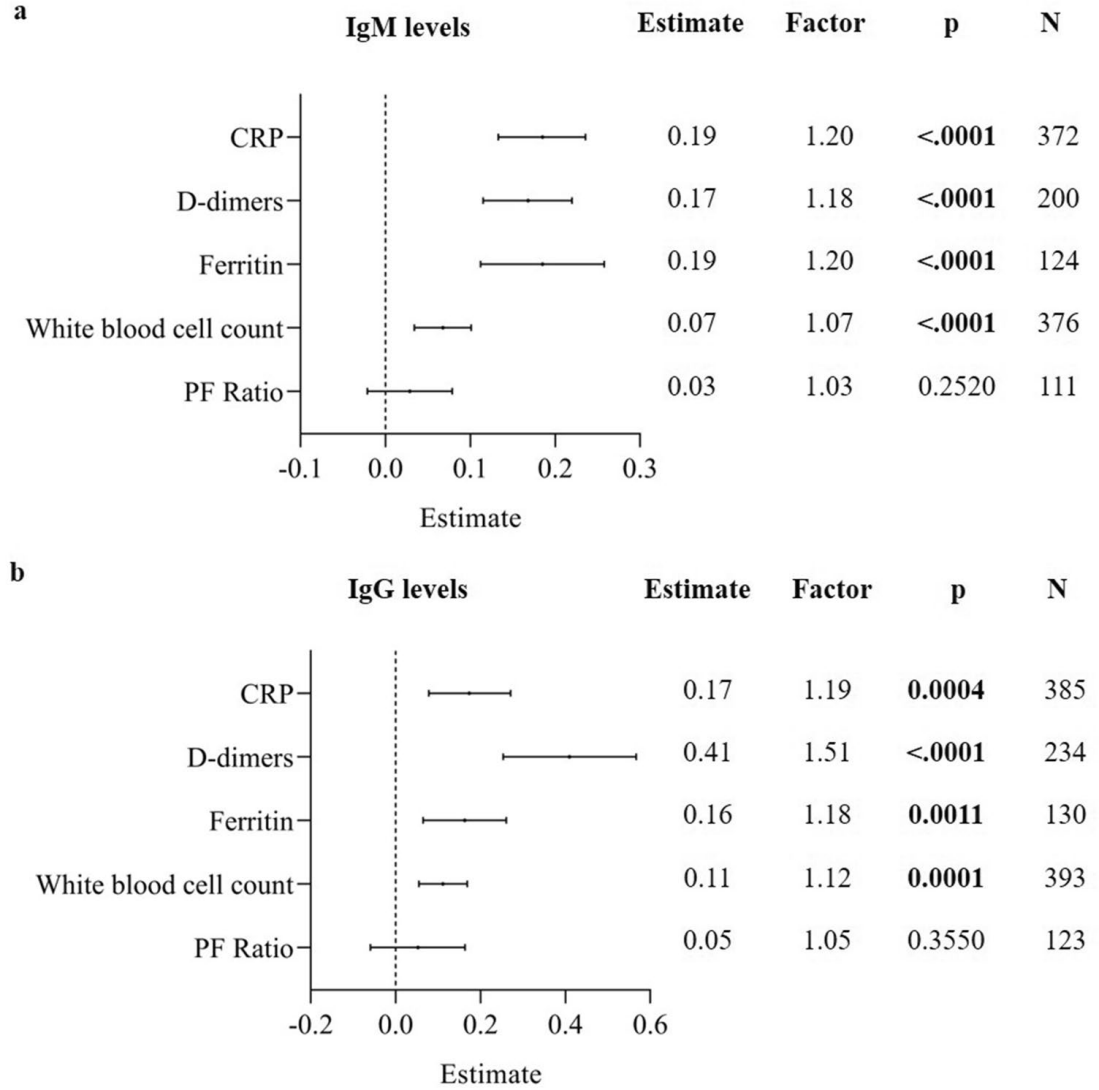
Association between baseline antibody levels and laboratory parameters. Results are shown as parameter estimates, which shows the average increase/decrease (on a natural log scale) of the corresponding marker with one unit increase on the log10 scale of the IgM (**a**) and IgG (**b**) antibody level using a forest plot. The factor indicates the change on the original scale with one unit increase on the log10 scale of the IgM/IgG antibody level. The parameter estimates were calculated using the adjusted GEE model. The models were adjusted through a backward model selection procedure including age, sex, BMI, comorbidities and therapies. Lines show 95% confidence intervals. N = number of patients included in the analysis. A p-value is significant if its value is lower than the level of significance, which is set at 5% and indicated in bold.

Increased levels of anti-SARS-CoV-2 IgG and IgM were positively associated with known soluble markers of inflammation including CRP, D-dimers, ferritin and the white blood cell count. Parameter estimates can be found in Fig. 3. Considering the log_10_ scale of the antibody levels and the natural log scale for the parameter estimate of the inflammatory variables, a tenfold increase of IgM levels was associated with an increase in the geometric mean of CRP (p < 0.001) and ferritin (p < 0.001) levels by a factor of 1.2, or by 20%. Moreover, tenfold higher IgG levels were associated an increase of 51% in the geometric mean of D-dimers (p < 0.001). No significant association was found between IgM and IgG antibody levels and the PF ratio.

### Baseline cytokine response in the Limburg COVID-19 cohort

**Figure 4:**
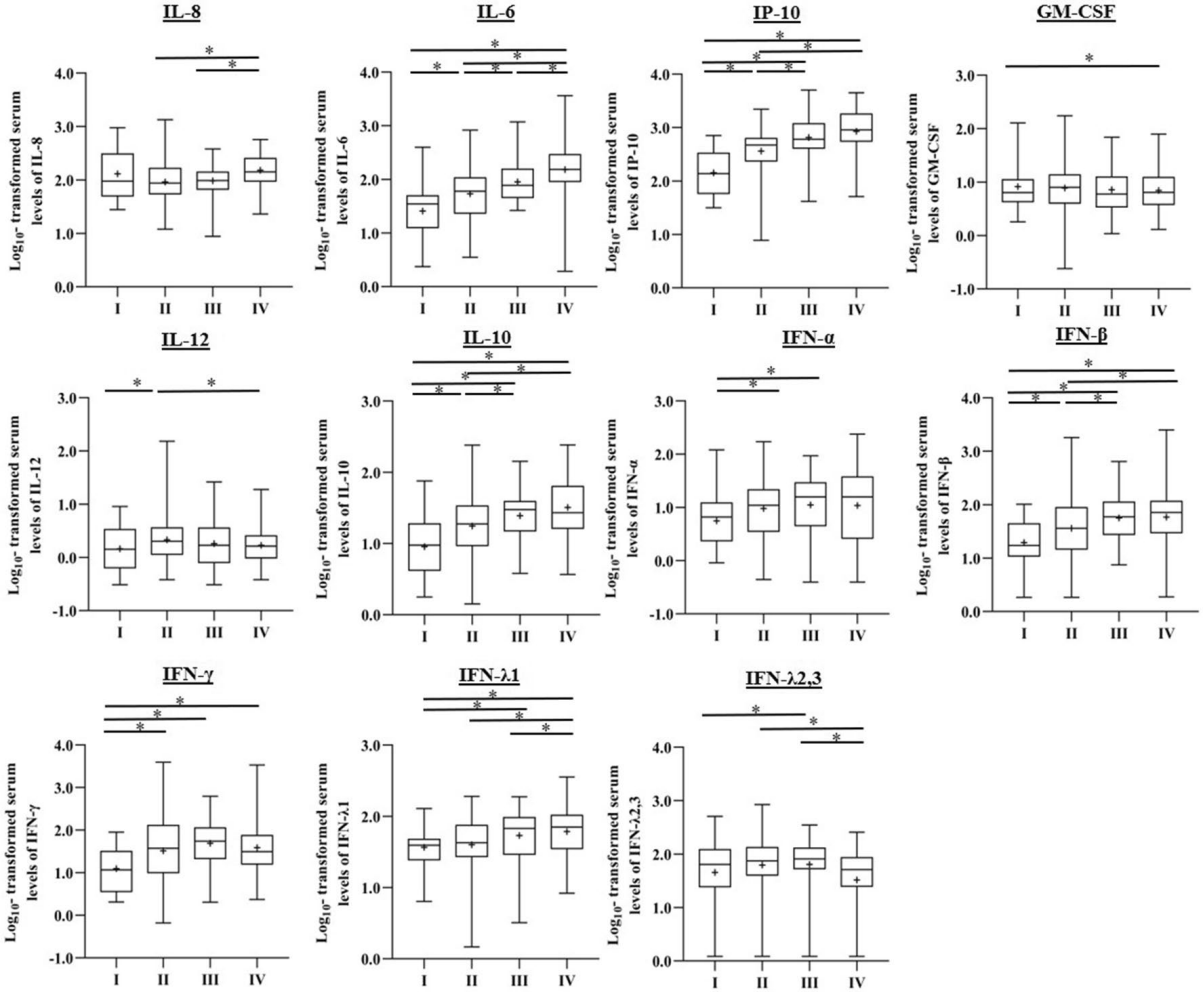
Cytokine levels in COVID-19 patients according to disease severity. Cytokine expression levels of 2 replicates per sample were measured in serum using Legendplex in the asymptomatic/mild (n = 35), moderate (n = 93), severe (n = 50) and critically-ill group (n = 55). The box plot visualizes the following summary statistics: the middle line represents the median; the lower hinge corresponds to the first quartile (25th); the upper hinge corresponds to the third quartile (75th); upper and lower whiskers extend from the hinge respectively to the largest value and smallest value. Data are shown as median (range), the diamond indicates the average level. Significance was calculated between the groups and *P-*values were adjusted for multiple comparisons using the Tuckey-Kramer method (*p < 0.05).

Cytokine profiles were measured in 246 COVID-19 patients. Serum concentrations of the pro-inflammatory mediators TNFα and IL-1β were below detection limit for about half of measured samples were therefore excluded from further analysis. Serum levels of the pro-inflammatory cytokines IL-6 and IP-10 and the anti-inflammatory cytokine IL-10 showed a stepwise increase according to disease severity (Fig. 4). For IL-8, a stepwise increase was observed in patients from the moderate towards the critically ill subgroup (Fig. 4). Remarkably, in our cohort, levels of the pro-inflammatory cytokine GM-CSF showed a decrease in critically ill patients compared to asymptomatic/mild patients (p = 0.0003). For IL-12, we found statistically different levels in patients with a moderate disease compared to the asymptomatic (p = 0.0011) and the critically ill group (p = 0.0419), observing higher levels in patients with moderate disease compared to asymptomatic or critically ill patients. Serum levels of the type I interferon cytokines (IFNα2 and IFNβ) and the type II interferon cytokine IFN-γ also increased with more severe COVID-19. Interestingly, levels of IFN-λ2,3 were significantly lower in critically ill patients compared to patients with a moderate (p < 0.0001) and severe disease (p < 0.0001), whereas IFN-λ1 levels progressively increased according to disease severity.

### Association between baseline cytokine levels and clinical outcome parameters

**Figure 5:**
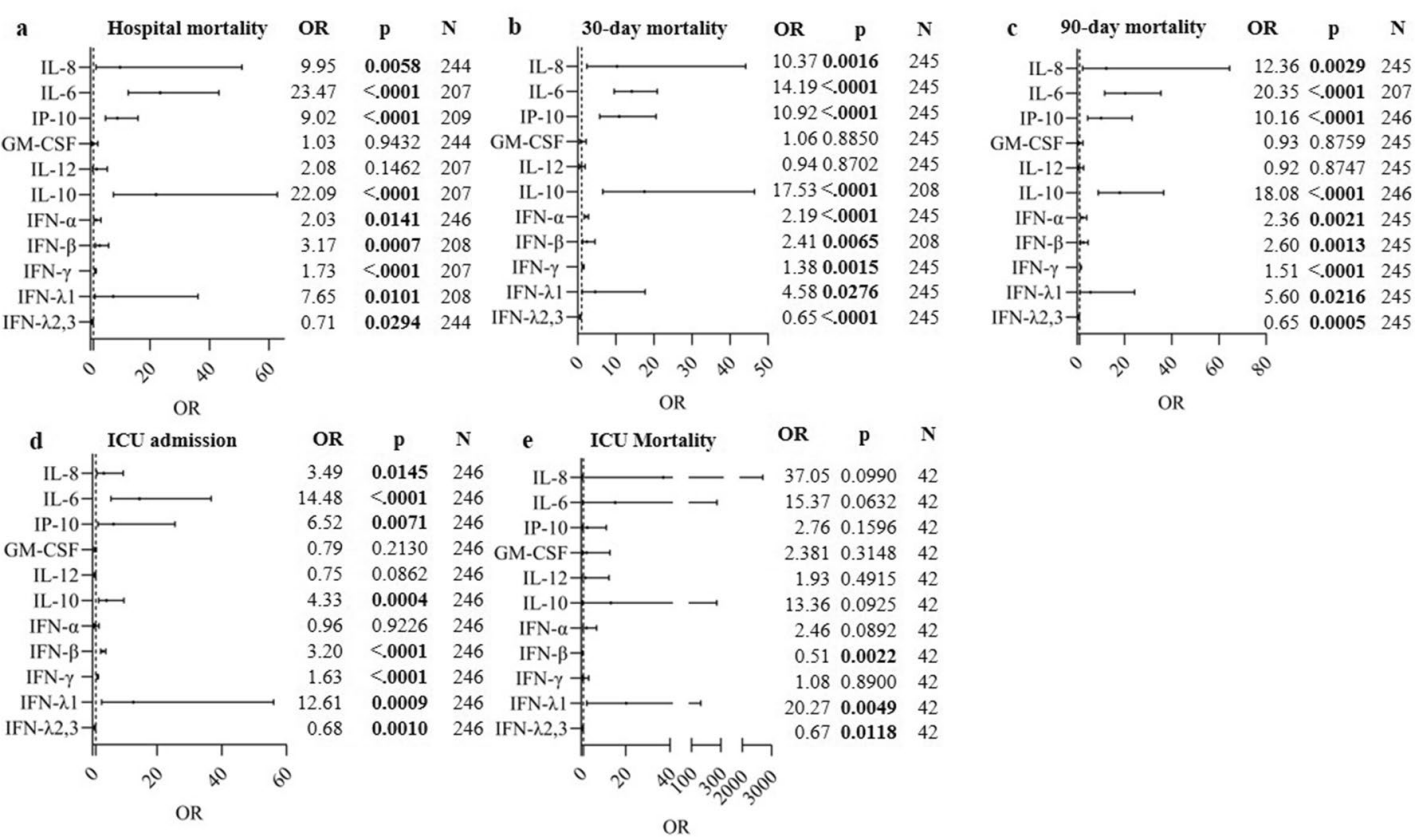
Association between baseline cytokine levels and clinical outcomes. Odds ratios (ORs) are shown for the association between cytokine levels and clinical outcomes including hospital mortality (**a**), 30-day mortality (**b**), 90-day mortality (**c**), ICU admission (**d**), and ICU mortality (**e**) using a forest plot. The ORs indicated in the table are calculated using the adjusted GEE model. The models were adjusted through a backward model selection procedure including sex, age, BMI, comorbidities and therapies. The OR indicated in the table is shown after adjustment for sex, age, BMI, comorbidities and therapies. Lines show 95% confidence intervals. N = number of patients included in the analysis. A p-value is significant if its value is lower than the level of significance, which is set at 5% and indicated in bold.

Increased baseline levels of 8 cytokines (IL-8, IL-6, IP-10, IL-10, IFN-α, IFN-β, IFN-γ and IFN-λ1) showed a statistically significant positive association with hospital mortality (Fig. 5a). Among these, the pro-inflammatory cytokine IL-6 (OR = 23.47, 95% CI 12.75–43.22, p < 0.0001), and the anti-inflammatory cytokine IL-10 (OR = 22.09, 95% CI 7.77–62.73, p < 0.0001), showed the highest increased odds for hospital mortality for a tenfold increase in baseline cytokine levels. Although a tenfold increase in baseline IFN-λ1 levels also resulted in an increase in the odds for hospital mortality (OR = 7.65, 95% CI 1.62–36.09, p = 0.0101), increasing IFN-λ2,3 levels at baseline were associated with lower odds for hospital mortality. More specifically, for a tenfold increase in IFN-λ2,3 levels, the odds for hospital mortality decreased with 29.3% (OR = 0.71, 95% CI 0.52–0.97, p = 0.0294). For all cytokines, similar results were obtained when considering 30-day mortality (Fig. 5b) and 90-day mortality (Fig. 5c) as clinical endpoint measurements. Furthermore, we found that increased levels of IL-8, IL-6, IP-10, IL-10, IFN-β, IFN-γ and IFN-λ1 were associated with increased odds for ICU admission (Fig. 5d). The highest ORs were found for IL-6 (OR = 14.48, 95% CI 5.71–36.73, p < 0.0001) and IFN-λ1 (OR = 12.6, 95% CI 2.8–56.1, p = 0.0009). On the other hand, increased levels of IFN-λ2,3 were associated with decreased odds for ICU admission (OR = 0.68, 95% CI 0.55–0.86, p = 0.001). For the subset of ICU patients (n = 42), the association between the pro-inflammatory cytokines (IL-6, IL-8, IP-10) and anti-inflammatory cytokine IL-10 levels and ICU mortality (Fig. 5e) was comparable to the association with hospital mortality of the entire Limburg COVID-19 cohort, but the majority of the associations were not significant. The OR for ICU mortality was significantly associated with IFN-λ1, resulting in a higher OR for ICU mortality in patients with higher levels of IFN-λ1 (OR = 20.27, 95% CI 2.49–164.76, p = 0.0049), while increased levels of IFN-λ2,3 (OR = 0.67, 95% CI 0.49–0.92, p = 0.0118) and IFN-β (OR = 0.51, 95% CI 0.33–0.78, p = 0.0022) levels were associated with a decrease in the odds of ICU mortality (Fig. 5e).

### Association between baseline cytokine levels and laboratory parameters

**Figure 6:**
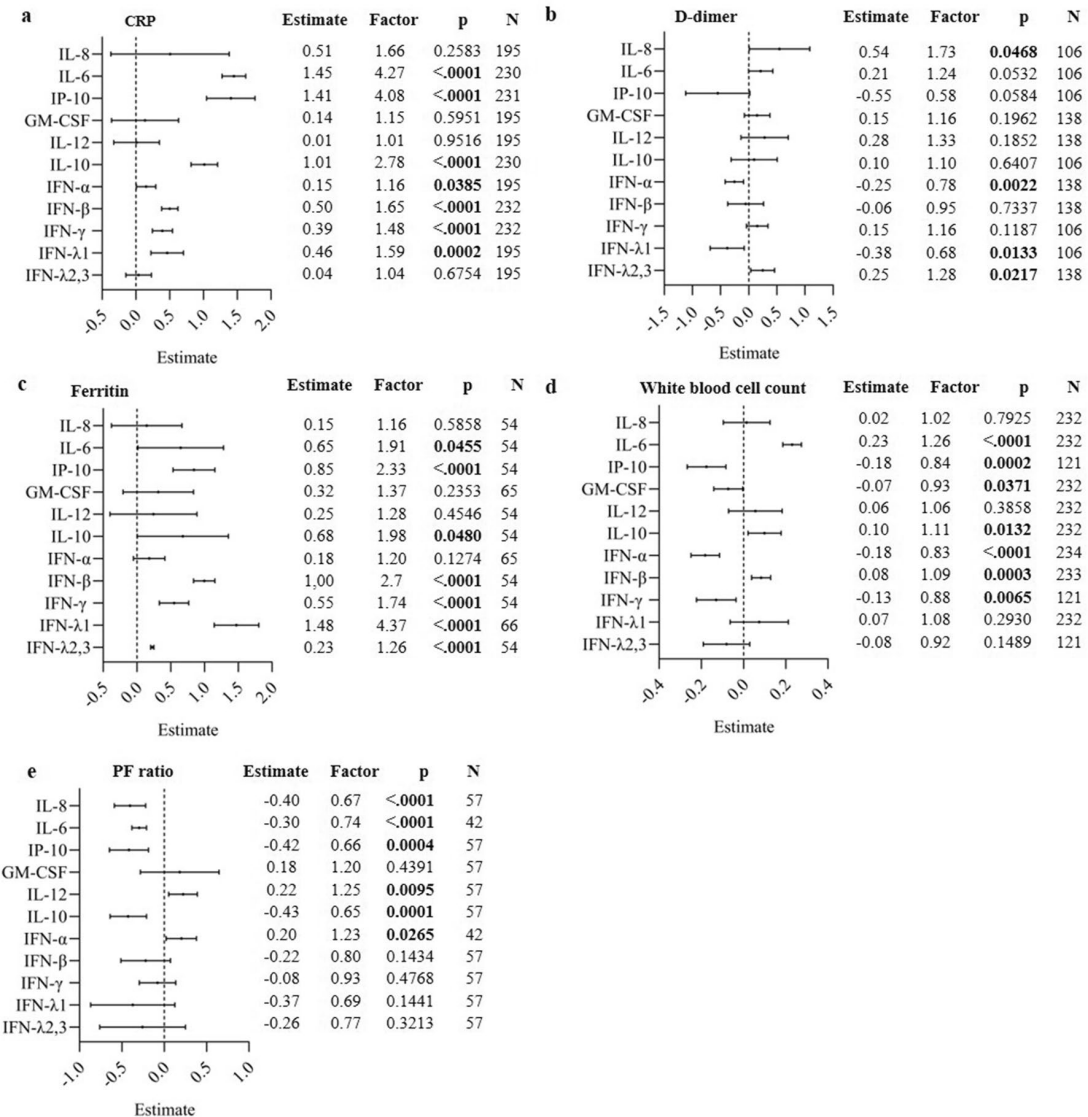
Association between baseline cytokine levels and laboratory parameters. Results are shown as parameter estimates, which shows the average increase/decrease (on a natural log scale) of the CRP (**a**), D-dimer (**b**), Ferritin (**c**), white blood cell count (**d**) and PF ratio (**e**) with one unit increase on the log10 scale of cytokine levels using a forest plot. The factor indicates the change on the original scale with one unit increase on the log10 scale of the cytokine level. The parameter estimates were calculated using the adjusted GEE model. The models were adjusted through a backward model selection procedure including age, sex, BMI, comorbidities and therapies. Lines show 95% confidence intervals. N = number of patients included in the analysis.

The association between the measured serum cytokines and the inflammatory markers CRP, D-dimers, ferritin and white blood-cell count and the clinical severity marker PF ratio at baseline were assessed and are shown in Fig. 6. All serum cytokines were positively associated with CRP (Fig. 6a), with significant associations for IL-6 (p < 0.0001), IP-10 (p < 0.0001), IL-10 (p < 0.0001), IFN-α (p = 0.0385), IFN-β (p < 0.0001), IFN-γ (p < 0.0001) and IFN-λ1 (p = 0.0002). The strongest association was found for IL-6 and IP-10, for which a tenfold increase in cytokine levels resulted in a fourfold increase in the geometric mean of CRP levels. Significant positive associations were found between IL-8 (p = 0.0468), IFN-λ2,3 (p = 0.0217), and D-dimers, whereas IFN-α (p = 0.0022) and IFN-λ1 (p = 0.0133) showed a negative association (Fig. 6b). Furthermore, a significant positive association was found between IL-6 (p = 0.0455), IP-10 (p < 0.0001), IL-10 (p = 0.048), IFN-β (p < 0.0001), IFN-γ (p < 0.0001), IFN-λ1 (p < 0.0001) and IFN-λ2,3 (p < 0.0001) and ferritin levels (Fig. 6c). The serum cytokines IL-6 (p < 0.0001), IL-10 (p = 0.0132) and IFN-β (p = 0.0003) were positively associated with the white blood cell count, whereas GM-CSF (p = 0.0371)**,** IP-10 (p = 0.0002), IFN-α (p < 0.0001) and IFN-γ (p = 0.0065) were negatively associated (Fig. 6d)**.** A negative association was found between IL-8 (p < 0.0001), IL-6 (p < 0.0001), IP-10 (p = 0.0004), IL-10 (p = 0.0001) levels and the PF ratio, with the most pronounced effect for IL-10. A tenfold increase of IL-10 levels resulted in a decrease of the geometric mean of PF ratio by 35% (Fig. 6e). In contrast, increasing IL-12 (p = 0.0095) and IFN-α (p = 0.0265) levels were positively associated with the PF ratio.

## Discussion

In this multi-centre cohort study, we characterized the antibody and cytokine responses in plasma and serum samples of respectively 411 and 246 hospitalised COVID-19 patients, obtained during the first wave of the COVID-19 pandemic in Belgium. The measured antibody and cytokine levels were linked to several clinical outcome parameters including hospital mortality, 30-day and 90-day mortality, ICU admission and ICU mortality and to different laboratory parameters. In this study, it was shown that baseline anti-spike protein IgM and IgG antibodies increased according to disease severity but were associated with decreased odds for hospital mortality. Furthermore, we showed that the pro-inflammatory cytokines (IL-8, IL-6, IP-10), the anti-inflammatory cytokine (IL-10) and the anti-viral cytokines (IFN-α, IFN-β, IFN-γ and IFN-λ1) at baseline were positively associated with hospital mortality, whereas higher IFN-λ2,3 levels were suggested to be protective against hospital mortality.

In previous studies, increasing age, male sex, and certain comorbidities were recognized as risk factors for poor outcomes of COVID-19^23–25^. In our cohort of 434 COVID-19 PCR-confirmed positive patients we found that men were more often severely affected by COVID-19 than women in all stages of the disease. Furthermore, obesity was observed as a major risk factor for adverse outcomes with a linear correlation between BMI and admission to ICU^26^. We also observed an increase in BMI in moderately, severely and critically ill patients compared to asymptomatic/mild patients. When analyzing laboratory parameters in our cohort, we found raised levels of CRP, ferritin and D-dimers according to disease severity. This is in line with previous findings, which already showed associations between these inflammatory markers and COVID-19 severity and progression^27^.

Although antibodies are critical in control of and protection from viral infections such as influenza^28^, the involvement of antibodies in SARS-CoV-2 clearance and modulation of COVID-19 disease severity remains to be precisely defined. In general, patients infected with SARS-CoV-2 generate virus-specific IgM, IgG and IgA antibodies, peaking between weeks 2 and 5 post-symptom onset^29^. In the Limburg COVID-19 cohort, anti-spike protein antibodies of the IgM and IgG isotype were detected at baseline in respectively 53.5% and 24.1% of patients. Considering the timing of IgM and IgG production in the natural course of an infection, this indicates that for about half of our cohort, the baseline sample was taken very early after the initial infection before the onset of a detectable IgM response. Similarly, these findings also suggest that about a quarter of included patients, who had already formed IgG antibodies, were hospitalized 2 to 3 weeks after SARS-CoV-2 infection. The currently available literature strongly suggests that the intensity of the IgM and IgG antibody response is associated with clinical severity of COVID-19^30–34^. In our patient cohort, we further corroborate these findings, as baseline levels of anti-spike IgM and IgG were higher in patients with a more severe disease. Moreover, the baseline antibody response was also associated with several inflammatory biomarkers of the host response, such as levels of CRP, ferritin and D-dimers. So far, it is not completely clear why antibody levels correlate with COVID-19 severity. However, as baseline anti-spike antibody levels are associated with levels of inflammatory biomarkers, it is most likely that this reflects a general immune response. It has already been shown previously that higher viral loads and stronger antibody responses are related to a more severe disease status^30, 35^. Unfortunately, cycle threshold (Ct) values, which semi-quantitatively assess the SARS-CoV-2 viral load, were not recorded for the present cohort. Furthermore, it has been found that the frequency of plasmablasts, the dividing antibody-secreting cells^36^, was increased according to disease severity^37, 38^. Alternatively, it could be suggested that antibody-dependent enhancement (ADE), the phenomenon by which antibodies can strengthen virus entry and replication, can play a role in COVID-19 severity, which was recently shown to occur in blood monocytes, inducing inflammatory cell death and possibly contributing to excessive release of pro-inflammatory cytokines^39^.

In contrast with the known association between antibody response and COVID-19 severity, the association between the antibody response and mortality in COVID-19 patients is largely underreported. In this respect, we found that increased IgM and IgG anti-spike antibody levels at baseline were associated with decreased odds for hospital, 30-day and 90-day mortality, indicating a protective role of anti-spike antibodies against mortality. Previous studies in small groups of critically ill patients have already shown that IgG and IgM anti-spike antibody titres were higher in COVID-19 patients who survived compared to those who did not^40, 41^, pointing towards a protective effect in critically ill patients. Considering the well-established association between antibody levels and severity, the data support the idea that the antibody response alone is insufficient to avoid a severe disease course, but a robust anti-spike antibody response can be essential to survive.

In line with previous studies, we found that the measured pro-inflammatory cytokines IL-6, IL-8 and IP-10 were higher in patients with a more severe COVID-19 disease category at baseline. Additionally, they were associated with increased odds for mortality. Indeed, it has been reported that IL-6, IL-8 and IP-10 levels are associated with increased severity and poor outcome, and IL-6 and IL-8 are considered as independent markers of severe COVID-19^15, 22^. Of the upregulated inflammatory cytokines, IL-6 has been considered as a key cytokine involved in the cytokine storm triggered by COVID-19 and given the high levels of this cytokine induced by SARS-CoV-2, IL-6 blocking agents have been used for treating severe COVID-19. So far, clinical studies testing these antagonists have shown various effects in patients with COVID-19^42^. Results from a meta-analysis indicate that treatment with tocilizumab, a humanized anti-IL-6 receptor antibody, reduces all-cause mortality at day-28, but little or no clinical improvement could be observed^43^. Furthermore, in the multicentre, open-label, randomised, controlled COV-AID trial, no clinical benefit was observed upon blockade of the IL-6 or IL-1 pathway early in the disease course of hypoxic COVID-19 patients with evidence of systemic cytokine release syndrome^44^. Additionally, reparixin, an IL-8 receptor inhibitor, is currently being tested in clinical trials for efficacy in hospitalized adult patients with moderate or severe COVID-19 pneumonia.

On the other hand, we found that an increase in IL-10 levels, which is known for its anti-inflammatory effects, was associated with increased mortality and risk for ICU admission. This is in line with previous studies reporting that IL-10 levels predict poor outcomes in patients with COVID-19^19, 45^. The strong increase of the anti-inflammatory cytokine IL-10 within the cytokine storm can be considered as a secondary, albeit counter regulatory response to pro-inflammatory cytokines^46^. Nevertheless, it must be noted that non-classical pro-inflammatory and immunostimulatory effects of IL-10 have been found in certain inflammatory conditions, such as COVID-19^45^. Given the ability of IL-10 to induce T cell activation in cancer models^47^, IL-10 levels have been found to correlate with IFN-γ producing CD4 + and CD8 + T cells^48^ and exhausted T cells^49^, suggesting a role of IL-10 in T cell exhaustion in COVID-19.

It remains unclear whether IFNs have protective or detrimental effects in COVID-19 patients. Several studies have reported that type I and III IFN responses in patients with severe COVID-19 are dampened during the early phase of infection^50, 51^, however other studies have shown that patients with severe COVID-19 have robust type I IFN responses^6, 9^. Both type I (IFN-α, IFN-β) and type III (IFN-λ1, IFN-λ2,3) IFNs share antiviral features, but type I IFNs induce a systemic pro-inflammatory response, whereas type III IFNs suppress viral spread without causing inflammation^52, 53^. In our study, progressively higher levels of IFN-α2 and IFN-β across the severity spectrum from asymptomatic/mild to critically ill patients were observed. Furthermore, a significant association was found between baseline IFN-α2 and IFN-β levels and hospital mortality, i.e. the odds ratio for hospital mortality was higher for patients with higher baseline IFN-α2 and IFN-β levels. Previously, high levels of IFN-α were reported to be strongly associated with disease severity at an early time point (before day 12 from symptom onset) and were associated with longer hospital stay and mortality^22^.

Remarkably, when further investigating the type III IFN response, IFN-λ1 levels showed a stepwise increase according to disease severity, whereas levels of IFN-λ2,3 were significantly lower in critically ill patients compared to patients with moderate and severe disease. Furthermore, our study reveals an opposite effect of increased IFNλ1 and IFNλ2,3 levels on hospital mortality. Current data regarding IFN-λ2,3 in serum are rather limited, but it must be noted that the significant decrease in IFN-λ2,3 was also observed in serum samples of severe COVID-19 patients from Japan^54^. These results together with the earlier mentioned unique biological properties of type III IFNs make them attractive therapeutic agents in COVID-19 patients. In vitro studies indeed demonstrate the suppression of SARS‐CoV‐2 replication in a mouse model by pegylated human IFN-λ1^55^. Additionally, SARS‐CoV‐2 RNA expression was decreased in primary human airway epithelial cells when pretreated with IFN-λ1^56^. Currently, clinical trials using pegylated IFN-λ1 in COVID-19 patients are ongoing. However, first results with a single subcutaneous injection of IFN-λ1 did not show a significantly reduced time of viral clearance or resolution of symptoms compared with placebo^57^. On the other hand, despite the well-known antiviral effects of type III IFNs, it has been recently shown that IFN-λ can disrupt the lung epithelial barrier in mice, leading to worsened disease score and increased susceptibility to bacterial superinfection^58, 59^. Moreover, in bronchoalveolar lavage fluid of severe COVID-19 patients elevated mRNA expression of inflammatory cytokines, as well as type I and III IFNs was found^59^. High expression of type I and III IFNs in the lung associated with COVID-19 disease morbidity^59^. Furthermore, IFN-λ has also been shown to inhibit influenza virus-stimulated B-cell activation and antibody production, thereby suggesting a negative effect on the adaptive immune response critical to resolution of infection^60^*.* Understanding the location, timing and duration are probably critical parameters of the interferon response and should be taken into account for IFN therapeutic strategies. Interestingly, both IFNλ1 and IFNλ2 are upregulated in patients infected with hepatitis C virus, but remarkably in in vitro studies IFN-λ2 but not IFN-λ1 acts as potent gene repressor^61^. The latter one suggests a possible explanation for the observed opposite effect of increased IFN-λ1 and IFN-λ2,3 levels on hospital mortality. Further studies are needed to find out whether the 3 members of the IFN-λ family cause different effects in COVID-19 disease.

Our study has several limitations. A potential source of bias in the results may stem from different timing of sampling for antibodies and cytokines in COVID-19 patients. The timing was chosen at 4 and 8 days after a positive PCR for antibodies and cytokines, respectively. Onset of symptoms was not considered due to its unreliable and often unavailable recording during the first wave of the COVID-19 pandemic. Furthermore, the wide confidence intervals that are associated to the ORs need to be interpreted with care. This issue is most likely the result of a lower sample size in certain subgroups.

As conclusion, our data indicate that baseline anti-spike IgM and IgG levels are associated with clinical severity, but are associated with decreased odds for hospital mortality. Furthermore, baseline levels of the pro-inflammatory cytokines IL-8, IL-6, and IP-10, the anti-inflammatory cytokine IL-10, and the anti-viral cytokines IFN-α, IFN-β, IFN-γ and IFN-λ1 are positively associated with mortality, whereas higher IFN-λ2,3 levels are suggested to be protective against mortality.

## Materials and methods

### Study design and patients

A retrospective cross-sectional study was performed using plasma and serum samples from 434 hospitalized COVID-19 patients with a confirmed nasopharyngeal swab PCR diagnosis in the hospital. Samples were collected in 5 different hospitals (Ziekenhuis Oost-Limburg (Genk), Jessa hospital (Hasselt), Noorderhart hospital (Pelt), Algemeen Ziekenhuis Vesalius (Tongeren) and St-Trudo hospital (Sint-Truiden)) in the province of Limburg in Belgium during the first wave of COVID-19 from March to June 2020, referred to as the Limburg COVID-19 cohort (3 patients included in the cohort were admitted between July 1 and September 9 2020). The human biological material used in this publication was stored by the University Biobank Limburg (UBiLim) at − 80 °C prior to analyses^62^. All serum and/or plasma samples were collected at baseline, which is within 4 days after a positive PCR for antibody detection and within 8 days after a positive PCR for cytokine measurement.

Demographic, clinical and laboratory parameters and clinical outcome data of the hospitalized patients were obtained from medical records (Table 1 and 3). Demographic characteristics include age, sex and body mass index (BMI). Laboratory parameters including levels of CRP, D-dimers, ferritin, white blood cell count and the respiratory parameter PF ratio (the ratio of arterial oxygen partial pressure (PaO_2_) to fractional inspired oxygen (FiO_2_)) were measured at the time of baseline blood sampling. Clinical parameters include the presence of comorbidities in the medical record (arterial hypertension, diabetes, kidney injury, malignancies, lung disease, heart failure, hepatic failure and immunocompromised status) and use of therapies such as antibacterial therapy, antiviral medication including lopinavir and remdesivir, antifungal therapy, anti-inflammatory therapy including interleukin (IL) inhibitors, corticosteroids and hydroxychloroquine, renal replacement therapy, vasopressor use and any other therapy. Clinical outcome data, including in-hospital mortality (all-cause mortality during hospital stay), 30-day mortality (all-cause mortality within 30 days after hospital admission), 90-day mortality (all-cause mortality within 90 days after hospital admission), intensive care unit (ICU) admission and ICU mortality were evaluated during the course of hospitalisation and patient follow up.

At the time of the baseline blood sampling, patients were classified into 4 severity categories (Table 2) based on clinical characteristics according to the WHO clinical management of COVID-19 interim guidance issued May 27 2020^63^.

Documented approval was obtained from all institutional Medical Ethics Committees with central positive advice granted by the Commissions for Medical Ethics of UHasselt (CME2020/040) and Ziekenhuis Oost-Limburg (20/0058R). According to the Belgian Act of 19 December 2008 on the usage of human body material for scientific research, parts 1–2 and 5–20 no informed consent was needed for this retrospective study. All methods were carried out in accordance the Belgian Act of 19 December 2008 for this retrospective study.

### Antibody detection

IgM antibodies against the receptor binding domain (RBD) in the S1 subunit of the SARS-CoV-2 spike protein, and IgG antibodies against the S1 subunit of the SARS-CoV-2 spike protein, were detected in serum or plasma samples using enzyme linked immunosorbent assays (ELISA) (IgM, Beijing Wantai Biological; IgG, Euroimmun), according to the manufacturer’s instructions. Samples were considered seropositive according to the cut-off of the respective ELISA kits. IgG and IgM antibody levels were quantified by linear interpolation using serial dilutions of a positive plasma sample, which was later converted to arbitrary units (AU)/mL using the Anti-SARS-CoV-2 Antibody Diagnostic Calibrant (20/162) from the National Institute for Biological Standards and Control (NIBSC) in the United Kingdom.

### Cytokine analysis

Circulating serum levels of cytokines were measured by the LegendPlex Human Anti-Virus Response Panel (13-plex) (740, 390, BioLegend, San Diego, USA) according to the manufacturer’s instructions with minor adjustments. These include the cytokines TNF-α, IL-1β, IL-8, IL-6, IP-10, GM-CSF, IL-12, IFN-α, IFN-β, IFN-γ, IFN-λ1, IFN-λ2,3 and IL-10. The assay was carried out in V-bottom 96-well plates and serum (12.5 µL) was thawed and diluted twofold with assay buffer before testing. Standards, mixed beads, detection antibodies and streptavidin-PE were prepared according to the manufacturer’s instructions and 12.5 µL of each reagent was used. All serum samples were tested in duplicate. Data were collected using a LSRFortessa flow cytometer (BD Biosciences) and analysed using LEGENDplex™ Data Analysis Software Suite (BioLegend). If a value was below the assay lower limit of detection, then the value of the detection limit was used. The average cytokine level was calculated for duplicate measurements.

### Statistical analysis

Descriptive statistics for continuous patient demographics, clinical characteristics and clinical outcomes are reported as median and interquartile range (IQR), while discrete characteristics and outcomes are summarized as counts and percentages. To examine differences in continuous patient demographics according to disease severity, when data are not normally distributed, Aligned Rank Transform was applied to the data. Differences in discrete patient demographics according to disease severity were examined using a Generalized Estimating Equation (GEE) model^64^. Aforementioned methods correct for the correlated nature of the data, in which patients are clustered within hospitals. P-values were corrected for multiple comparisons according to the Tukey–Kramer method.

In order to examine the effect on antibody and cytokine response at baseline of disease severity, a GEE model was applied, which considers the correlation between responses of patients at the same hospital. For the analysis, antibody and cytokine levels were log_10_-transformed. Comparisons between levels of disease severity (category asymptomatic/mild, moderate, severe) were made using patients with a critically ill disease at baseline as a reference group.

The association between antibody or cytokine levels and clinical outcomes was investigated using a GEE model^64^, accounting for the correlation in the data. For the analysis, antibody and cytokine levels were log_10_-transformed, while for continuous outcome measures a natural logarithmic (ln) transformation was used. A backward model selection procedure was performed using patient’s age, sex, BMI, comorbidities and therapies for each clinical outcome parameter. Each final model only includes significant parameters and the term related to the antibody or cytokine levels (referred to as the adjusted model). The following covariates were included in the backward model selection procedure: age, BMI, sex, diabetes, arterial hypertension, kidney injury, lung disease, malignancies, immune status, antibacterial therapy ever, corticosteroid therapy ever, hydroxychloroquine and other therapy ever. This manuscript will only focus on result interpretations related to cytokine or antibody levels included in the adjusted model. However, the information on other significant covariates included in the adjusted model is available in the supplementary file. When studying the association between the cytokines and ICU admission and ICU mortality, an adjusted model was used only including age and sex as covariates in addition to the terms related to antibody or cytokine levels, no model selection was carried out since the models in the model selection procedure would not converge due to the small number of patients admitted at the ICU.

For binary outcome measures, the result of the adjusted GEE model is presented as the odds ratio (OR) for a one-unit increase of log_10_ antibody or cytokine levels. For continuous outcome measures, the result is presented as an average (denoted as parameter estimate), representing the increase or decrease of the ln of the outcome measure for a one-unit increase of log_10_ antibody or cytokine levels. Alternatively, the results of the adjusted GEE model for laboratory parameters are also presented as a factor representing the fold change of the geometric mean of the outcome (due to the back-transformation of a natural logarithm), for a tenfold increase in cytokine or antibody levels (a tenfold increase on the original scale corresponds to a one-unit increase on the log_10_ scale). The 95% confidence interval (CI) and p-value for each OR or parameter estimate were tabulated. P < 0.05 was deemed statistically significant. For all analysis performed, we assume that missing data are missing completely at random (Supplementary Information).

The statistical analyses were performed using SAS software for Windows, Version 9.4. (Copyright ©2016) and R (version 4.1.2)^65^.

### Supplementary Information

Supplementary Information.

## Data Availability

The raw data supporting the conclusions of this article will be made available by the authors, without undue reservation.
