# Supplementary material for: Comprehensive antibody and cytokine profiling in hospitalized COVID-19 patients in relation to clinical outcomes in a large Belgian cohort
Source: Sci Rep. 2023 Nov 7;13:19322. doi: 10.1038/s41598-023-46421-4 (PMC10630327; doi:10.1038/s41598-023-46421-4)
Supplement: Supplementary file 1 — Supplementary Information. [file 41598_2023_46421_MOESM1_ESM.zip › Adjusted GEE model for 30-day mortality with AB.pdf]

Adjusted GEE model for Mortality_30d with IgG_sero

Obs	Parm	Estimate	Stderr	LowerCL	UpperCL	Z	ProbZ
1	Intercept	-6.7762	0.6102	-7.9722	-5.5802	-11.10	<.0001
2	IgG_sero	-0.5357	0.2922	-1.1084	0.0370	-1.83	0.0667
3	Age	0.0587	0.0071	0.0449	0.0726	8.32	<.0001
4	antibacterial_ever	1.2817	0.2018	0.8860	1.6773	6.35	<.0001
5	diabetes	-0.8116	0.2776	-1.3556	-0.2676	-2.92	0.0035
6	gender2	-0.3390	0.1335	-0.6006	-0.0774	-2.54	0.0111
7	kidney_injury	0.5139	0.1903	0.1410	0.8869	2.70	0.0069
8	other_therapy_ever	0.2268	0.0670	0.0955	0.3582	3.39	0.0007

Adjusted GEE model for Mortality_30d with IgM_sero

Obs	Parm	Estimate	Stderr	LowerCL	UpperCL	Z	ProbZ
1	Intercept	-6.8728	0.7996	-8.4399	-5.3057	-8.60	<.0001
2	IgM_sero	-0.7068	0.2003	-1.0994	-0.3142	-3.53	0.0004
3	Age	0.0580	0.0082	0.0420	0.0741	7.10	<.0001
4	antibacterial_ever	1.4585	0.1602	1.1446	1.7724	9.11	<.0001
5	arterial_hypertension	0.5895	0.1827	0.2313	0.9476	3.23	0.0013
6	diabetes	-0.7755	0.2837	-1.3315	-0.2195	-2.73	0.0063

Adjusted GEE model for Mortality_30d with log10IgG

Obs	Parm	Estimate	Stderr	LowerCL	UpperCL	Z	ProbZ
1	Intercept	-6.8886	0.6757	-8.2129	-5.5642	-10.19	<.0001
2	IgG_NIBSC_avg	-0.5315	0.1120	-0.7510	-0.3120	-4.75	<.0001
3	Age	0.0596	0.0068	0.0463	0.0730	8.74	<.0001
4	antibacterial_ever	1.4636	0.2010	1.0696	1.8576	7.28	<.0001
5	arterial_hypertension	0.4910	0.1834	0.1315	0.8504	2.68	0.0074
6	diabetes	-0.8098	0.2674	-1.3338	-0.2858	-3.03	0.0025
7	gender2	-0.2636	0.1221	-0.5030	-0.0242	-2.16	0.0309

Adjusted GEE model for Mortality_30d   with log10IgM

Obs	Parm	Estimate	Stderr	LowerCL	UpperCL	Z	ProbZ
1	Intercept	-6.8554	0.8385	-8.4988	-5.2120	-8.18	<.0001
2	IgM_NIBSC_avg	-0.2418	0.0538	-0.3473	-0.1362	-4.49	<.0001
3	Age	0.0579	0.0087	0.0408	0.0750	6.63	<.0001
4	antibacterial_ever	1.4596	0.1799	1.1070	1.8122	8.11	<.0001
5	arterial_hypertension	0.5594	0.1621	0.2418	0.8770	3.45	0.0006
6	diabetes	-0.7459	0.2907	-1.3155	-0.1762	-2.57	0.0103
