# Supplementary material for: Comprehensive antibody and cytokine profiling in hospitalized COVID-19 patients in relation to clinical outcomes in a large Belgian cohort
Source: Sci Rep. 2023 Nov 7;13:19322. doi: 10.1038/s41598-023-46421-4 (PMC10630327; doi:10.1038/s41598-023-46421-4)
Supplement: Supplementary file 1 — Supplementary Information. [file 41598_2023_46421_MOESM1_ESM.zip › Adjusted GEE model for 30-day mortality with CYT.pdf]

Adjusted GEE model mortality_30d with log10IFNl1

Obs	Parm	Estimate	Stderr	LowerCL	UpperCL	Z	ProbZ
1	Intercept	-9.7547	1.4977	-12.6902	-6.8192	-6.51	<.0001
2	log10IFNL1	1.5205	0.6904	0.1673	2.8738	2.20	0.0276
3	Age	0.0567	0.0026	0.0517	0.0618	21.99	<.0001
4	antibacterial_ever	1.3264	0.1984	0.9375	1.7152	6.69	<.0001
5	arterial_hypertension	0.5717	0.1938	0.1918	0.9516	2.95	0.0032
6	kidney_injury	0.7260	0.3239	0.0911	1.3609	2.24	0.0250

Adjusted GEE model mortality_30d with log10IFNa

Obs	Parm	Estimate	Stderr	LowerCL	UpperCL	Z	ProbZ
1	Intercept	-8.0943	0.5021	-9.0785	-7.1101	-16.12	<.0001
2	log10IFNa	0.7828	0.1285	0.5308	1.0347	6.09	<.0001
3	Age	0.0583	0.0022	0.0541	0.0626	26.96	<.0001
4	antibacterial_ever	1.3440	0.2635	0.8274	1.8605	5.10	<.0001
5	arterial_hypertension	0.8087	0.2380	0.3423	1.2752	3.40	0.0007

Adjusted GEE model mortality_30d with log10IFNb

Obs	Parm	Estimate	Stderr	LowerCL	UpperCL	Z	ProbZ
1	Intercept	-8.5407	0.8915	-10.2880	-6.7935	-9.58	<.0001
2	log10IFNb	0.8781	0.3224	0.2463	1.5100	2.72	0.0065
3	Age	0.0541	0.0043	0.0457	0.0625	12.61	<.0001
4	antibacterial_ever	1.1891	0.2545	0.6902	1.6879	4.67	<.0001
5	arterial_hypertension	0.7932	0.2565	0.2905	1.2959	3.09	0.0020
6	kidney_injury	0.6933	0.2286	0.2453	1.1413	3.03	0.0024
7	other_therapy_ever	0.2893	0.1318	0.0309	0.5477	2.19	0.0282

Adjusted GEE model mortality_30d with log10IFNg

Obs	Parm	Estimate	Stderr	LowerCL	UpperCL	Z	ProbZ
1	Intercept	-7.6209	0.4307	-8.4650	-6.7767	-17.69	<.0001
2	log10IFNg	0.3222	0.1017	0.1228	0.5216	3.17	0.0015
3	Age	0.0569	0.0031	0.0509	0.0630	18.49	<.0001
4	antibacterial_ever	1.2545	0.2205	0.8223	1.6868	5.69	<.0001
5	arterial_hypertension	0.6871	0.2156	0.2646	1.1095	3.19	0.0014
6	kidney_injury	0.5671	0.2635	0.0506	1.0837	2.15	0.0314

Adjusted GEE model mortality_30d with log10IFNl23

Obs	Parm	Estimate	Stderr	LowerCL	UpperCL	Z	ProbZ
1	Intercept	-6.3084	0.2550	-6.8082	-5.8085	-24.73	<.0001
2	log10IFNl23	-0.4386	0.0758	-0.5872	-0.2899	-5.78	<.0001
3	Age	0.0564	0.0031	0.0504	0.0624	18.48	<.0001
4	antibacterial_ever	1.2035	0.2436	0.7261	1.6808	4.94	<.0001
5	arterial_hypertension	0.6710	0.1900	0.2985	1.0435	3.53	0.0004
6	kidney_injury	0.6469	0.2291	0.1979	1.0960	2.82	0.0047

Adjusted GEE model mortality_30d with log10IL10

Obs	Parm	Estimate	Stderr	LowerCL	UpperCL	Z	ProbZ
1	Intercept	-13.1038	0.8827	-14.8339	-11.3738	-14.85	<.0001
2	log10IL10	2.8639	0.4972	1.8894	3.8383	5.76	<.0001
3	Age	0.0852	0.0040	0.0774	0.0930	21.42	<.0001
4	antibacterial_ever	0.7676	0.2294	0.3180	1.2172	3.35	0.0008
5	gender2	-0.3563	0.0963	-0.5450	-0.1677	-3.70	0.0002
6	kidney_injury	0.8575	0.3084	0.2530	1.4619	2.78	0.0054
7	other_therapy_ever	0.8389	0.1322	0.5798	1.0981	6.35	<.0001

Adjusted GEE model mortality_30d with log10IL12

Obs	Parm	Estimate	Stderr	LowerCL	UpperCL	Z	ProbZ
1	Intercept	-6.9584	0.2656	-7.4790	-6.4378	-26.20	<.0001
2	log10IL12	-0.0607	0.3711	-0.7880	0.6667	-0.16	0.8702
3	Age	0.0545	0.0027	0.0493	0.0597	20.40	<.0001
4	antibacterial_ever	1.2743	0.2241	0.8352	1.7135	5.69	<.0001
5	arterial_hypertension	0.7074	0.2019	0.3117	1.1031	3.50	0.0005
6	kidney_injury	0.6443	0.2505	0.1535	1.1352	2.57	0.0101

Adjusted GEE model mortality_30d with log10IL6

Obs	Parm	Estimate	Stderr	LowerCL	UpperCL	Z	ProbZ
1	Intercept	-13.2662	0.2636	-13.7828	-12.7496	-50.33	<.0001
2	log10IL6	2.6526	0.1984	2.2637	3.0414	13.37	<.0001
3	Age	0.0800	0.0028	0.0745	0.0856	28.18	<.0001
4	antibacterial_ever	0.5038	0.0980	0.3117	0.6958	5.14	<.0001
5	kidney_injury	0.5631	0.1544	0.2605	0.8657	3.65	0.0003

Adjusted GEE model mortality_30d with log10IL8

Obs	Parm	Estimate	Stderr	LowerCL	UpperCL	Z	ProbZ
1	Intercept	-13.3327	2.7728	-18.7672	-7.8981	-4.81	<.0001
2	log10IL8	2.3393	0.7393	0.8903	3.7883	3.16	0.0016
3	Age	0.0674	0.0122	0.0435	0.0913	5.53	<.0001
4	antibacterial_ever	1.0493	0.2162	0.6256	1.4731	4.85	<.0001
5	arterial_hypertension	0.9514	0.2957	0.3718	1.5309	3.22	0.0013
6	hydroxychloroquine_ever	0.7078	0.2337	0.2498	1.1658	3.03	0.0025
7	kidney_injury	0.8367	0.1244	0.5930	1.0805	6.73	<.0001

Adjusted GEE model mortality_30d with log10IP10

Obs	Parm	Estimate	Stderr	LowerCL	UpperCL	Z	ProbZ
1	Intercept	-13.9900	0.6914	-15.3450	-12.6350	-20.24	<.0001
2	log10IP10	2.3903	0.3233	1.7566	3.0240	7.39	<.0001
3	Age	0.0667	0.0030	0.0609	0.0726	22.31	<.0001
4	antibacterial_ever	0.8771	0.0984	0.6842	1.0700	8.91	<.0001
5	arterial_hypertension	0.3854	0.1649	0.0621	0.7087	2.34	0.0195

Adjusted GEE model mortality_30d with log10GM

Obs	Parm	Estimate	Stderr	LowerCL	UpperCL	Z	ProbZ
1	Intercept	-7.0554	0.5307	-8.0957	-6.0152	-13.29	<.0001
2	log10GM	0.0539	0.3730	-0.6770	0.7849	0.14	0.8850
3	Age	0.0550	0.0042	0.0467	0.0633	13.00	<.0001
4	antibacterial_ever	1.2769	0.2261	0.8338	1.7201	5.65	<.0001
5	arterial_hypertension	0.7062	0.2254	0.2644	1.1480	3.13	0.0017
6	kidney_injury	0.6412	0.2605	0.1306	1.1517	2.46	0.0138
