# Supplementary material for: Comprehensive antibody and cytokine profiling in hospitalized COVID-19 patients in relation to clinical outcomes in a large Belgian cohort
Source: Sci Rep. 2023 Nov 7;13:19322. doi: 10.1038/s41598-023-46421-4 (PMC10630327; doi:10.1038/s41598-023-46421-4)
Supplement: Supplementary file 1 — Supplementary Information. [file 41598_2023_46421_MOESM1_ESM.zip › Adjusted GEE model for 90-day mortality with AB.pdf]

Adjusted GEE model for mortality_90d with IgG_sero

Obs	Parm	Estimate	Stderr	LowerCL	UpperCL	Z	ProbZ
1	Intercept	-5.9929	0.5632	-7.0968	-4.8890	-10.64	<.0001
2	IgG_sero	-0.3891	0.4595	-1.2898	0.5116	-0.85	0.3972
3	Age	0.0652	0.0064	0.0527	0.0777	10.24	<.0001
4	corticosteroids_ever	0.6193	0.1681	0.2897	0.9488	3.68	0.0002
5	diabetes	-0.4484	0.1752	-0.7918	-0.1050	-2.56	0.0105
6	gender2	-0.4023	0.1561	-0.7082	-0.0964	-2.58	0.0099

Adjusted GEE model for mortality_90d with IgM_sero

Obs	Parm	Estimate	Stderr	LowerCL	UpperCL	Z	ProbZ
1	Intercept	-6.5449	0.9036	-8.3160	-4.7738	-7.24	<.0001
2	IgM_sero	-0.6968	0.2668	-1.2196	-0.1740	-2.61	0.0090
3	Age	0.0640	0.0092	0.0460	0.0820	6.95	<.0001
4	antibacterial_ever	0.9212	0.2850	0.3627	1.4797	3.23	0.0012
5	corticosteroids_ever	0.4601	0.1817	0.1039	0.8162	2.53	0.0113
6	diabetes	-0.5159	0.2129	-0.9332	-0.0987	-2.42	0.0154

Adjusted GEE model for mortality_90d with log10IgG

Obs	Parm	Estimate	Stderr	LowerCL	UpperCL	Z	ProbZ
**1**	Intercept	-6.5765	0.7562	-8.0587	-5.0944	-8.70	<.0001
**2**	IgG_NIBSC_avg	-0.4355	0.1394	-0.7087	-0.1623	-3.12	0.0018
**3**	Age	0.0655	0.0064	0.0529	0.0780	10.21	<.0001
**4**	antibacterial_ever	0.9026	0.2993	0.3160	1.4893	3.02	0.0026
**5**	corticosteroids_ever	0.4397	0.1669	0.1125	0.7668	2.63	0.0084
**6**	diabetes	-0.5534	0.1934	-0.9325	-0.1743	-2.86	0.0042
**7**	gender2	-0.3544	0.1625	-0.6729	-0.0360	-2.18	0.0291

Adjusted GEE model for mortality_90d with log10IgM

Obs	Parm	Estimate	Stderr	LowerCL	UpperCL	Z	ProbZ
1	Intercept	-6.5484	0.9688	-8.4472	-4.6495	-6.76	<.0001
2	IgM_NIBSC_avg	-0.2186	0.0856	-0.3864	-0.0509	-2.55	0.0106
3	Age	0.0638	0.0101	0.0440	0.0836	6.30	<.0001
4	antibacterial_ever	0.9138	0.2757	0.3733	1.4542	3.31	0.0009
5	corticosteroids_ever	0.4327	0.1893	0.0617	0.8037	2.29	0.0222
6	diabetes	-0.4945	0.2131	-0.9122	-0.0768	-2.32	0.0203
