# Supplementary material for: Comprehensive antibody and cytokine profiling in hospitalized COVID-19 patients in relation to clinical outcomes in a large Belgian cohort
Source: Sci Rep. 2023 Nov 7;13:19322. doi: 10.1038/s41598-023-46421-4 (PMC10630327; doi:10.1038/s41598-023-46421-4)
Supplement: Supplementary file 1 — Supplementary Information. [file 41598_2023_46421_MOESM1_ESM.zip › Adjusted GEE model for 90-day mortality with CYT.pdf]

Adjusted GEE model mortality_90d with log10IFNl1

Obs	Parm	Estimate	Stderr	LowerCL	UpperCL	Z	ProbZ
1	Intercept	-8.3499	1.3758	-11.0465	-5.6533	-6.07	<.0001
2	log10IFNL1	1.7235	0.7503	0.2529	3.1940	2.30	0.0216
3	Age	0.0485	0.0069	0.0350	0.0620	7.03	<.0001
4	arterial_hypertension	0.6968	0.1758	0.3522	1.0415	3.96	<.0001
5	corticosteroids_ever	0.9529	0.3652	0.2372	1.6687	2.61	0.0091

Adjusted GEE model mortality_90d with log10IFNa

Obs	Parm	Estimate	Stderr	LowerCL	UpperCL	Z	ProbZ
1	Intercept	-6.2794	0.8616	-7.9681	-4.5906	-7.29	<.0001
2	log10IFNa	0.8598	0.2791	0.3128	1.4068	3.08	0.0021
3	Age	0.0478	0.0063	0.0355	0.0602	7.61	<.0001
4	arterial_hypertension	0.8753	0.2295	0.4255	1.3251	3.81	0.0001
5	corticosteroids_ever	0.9488	0.3101	0.3410	1.5566	3.06	0.0022

Adjusted GEE model mortality_90d with log10IFNb

Obs	Parm	Estimate	Stderr	LowerCL	UpperCL	Z	ProbZ
1	Intercept	-6.8612	0.5477	-7.9347	-5.7877	-12.53	<.0001
2	log10IFNb	0.9566	0.2977	0.3730	1.5401	3.21	0.0013
3	Age	0.0468	0.0071	0.0329	0.0607	6.60	<.0001
4	arterial_hypertension	0.7730	0.2155	0.3506	1.1954	3.59	0.0003
5	corticosteroids_ever	0.7364	0.3490	0.0523	1.4205	2.11	0.0349

Adjusted GEE model mortality_90d with log10IFNg

Obs	Parm	Estimate	Stderr	LowerCL	UpperCL	Z	ProbZ
1	Intercept	-6.1007	0.6489	-7.3726	-4.8289	-9.40	<.0001
2	log10IFNg	0.4141	0.0355	0.3446	0.4837	11.67	<.0001
3	Age	0.0494	0.0069	0.0358	0.0630	7.11	<.0001
4	arterial_hypertension	0.7896	0.2282	0.3424	1.2368	3.46	0.0005
5	corticosteroids_ever	0.8884	0.3091	0.2826	1.4941	2.87	0.0040

Adjusted GEE model mortality_90d with log10IFNl23

Obs	Parm	Estimate	Stderr	LowerCL	UpperCL	Z	ProbZ
1	Intercept	-4.6425	0.6458	-5.9083	-3.3767	-7.19	<.0001
2	log10IFNl23	-0.4263	0.1225	-0.6663	-0.1863	-3.48	0.0005
3	Age	0.0478	0.0068	0.0345	0.0610	7.07	<.0001
4	arterial_hypertension	0.8194	0.1884	0.4502	1.1885	4.35	<.0001
5	corticosteroids_ever	0.9006	0.3308	0.2522	1.5489	2.72	0.0065

Adjusted GEE model mortality_90 with log10IL10

Obs	Parm	Estimate	Stderr	LowerCL	UpperCL	Z	ProbZ
1	Intercept	-5.3840	0.4469	-6.2598	-4.5081	-12.05	<.0001
2	log10IL10	2.8948	0.3599	2.1895	3.6002	8.04	<.0001

Adjusted GEE model mortality_90d with log10IL12

Obs	Parm	Estimate	Stderr	LowerCL	UpperCL	Z	ProbZ
1	Intercept	-5.2308	0.5916	-6.3902	-4.0714	-8.84	<.0001
2	log10IL12	-0.0893	0.5662	-1.1989	1.0204	-0.16	0.8747
3	Age	0.0462	0.0064	0.0337	0.0587	7.24	<.0001
4	arterial_hypertension	0.8441	0.2086	0.4353	1.2530	4.05	<.0001
5	corticosteroids_ever	0.9103	0.3435	0.2370	1.5835	2.65	0.0080

Adjusted GEE model mortality_90d with log10IL6

Obs	Parm	Estimate	Stderr	LowerCL	UpperCL	Z	ProbZ
1	Intercept	-14.0660	0.1194	-14.3000	-13.8320	-117.82	<.0001
2	log10IL6	3.0129	0.2833	2.4576	3.5681	10.63	<.0001
3	Age	0.0845	0.0077	0.0695	0.0995	11.02	<.0001
4	diabetes	-0.5597	0.2501	-1.0499	-0.0695	-2.24	0.0252
5	kidney_injury	0.5322	0.1465	0.2451	0.8192	3.63	0.0003
6	lung_disease	1.0461	0.4303	0.2027	1.8894	2.43	0.0151
7	other_therapy_ever	0.5091	0.1666	0.1826	0.8357	3.06	0.0022

Adjusted GEE model mortality_90d with log10IL8

Obs	Parm	Estimate	Stderr	LowerCL	UpperCL	Z	ProbZ
1	Intercept	-11.9788	2.8045	-17.4754	-6.4822	-4.27	<.0001
2	log10IL8	2.5142	0.8432	0.8615	4.1669	2.98	0.0029
3	Age	0.0569	0.0138	0.0298	0.0840	4.12	<.0001
4	arterial_hypertension	0.9897	0.2579	0.4844	1.4951	3.84	0.0001
5	corticosteroids_ever	0.7665	0.3777	0.0261	1.5068	2.03	0.0424
6	hydroxychloroquine_ever	0.6959	0.3251	0.0587	1.3332	2.14	0.0323
7	kidney_injury	0.8652	0.2064	0.4606	1.2698	4.19	<.0001

Adjusted GEE model mortality_90d with log10IP10

Obs	Parm	Estimate	Stderr	LowerCL	UpperCL	Z	ProbZ
1	Intercept	-7.6669	1.1488	-9.9184	-5.4153	-6.67	<.0001
2	log10IP10	2.3185	0.4222	1.4910	3.1461	5.49	<.0001

Adjusted GEE model mortality_90d with log10GM

Obs	Parm	Estimate	Stderr	LowerCL	UpperCL	Z	ProbZ
1	Intercept	-5.1725	0.8181	-6.7760	-3.5689	-6.32	<.0001
2	log10GM	-0.0783	0.5015	-1.0612	0.9046	-0.16	0.8759
3	Age	0.0460	0.0068	0.0327	0.0593	6.77	<.0001
4	arterial_hypertension	0.8461	0.2246	0.4059	1.2863	3.77	0.0002
5	corticosteroids_ever	0.9085	0.3369	0.2482	1.5687	2.70	0.0070
