# Supplementary material for: Comprehensive antibody and cytokine profiling in hospitalized COVID-19 patients in relation to clinical outcomes in a large Belgian cohort
Source: Sci Rep. 2023 Nov 7;13:19322. doi: 10.1038/s41598-023-46421-4 (PMC10630327; doi:10.1038/s41598-023-46421-4)
Supplement: Supplementary file 1 — Supplementary Information. [file 41598_2023_46421_MOESM1_ESM.zip › Adjusted GEE model for hospital mortality with AB.pdf]

Adjusted GEE model for Hospital mortality with IgG_sero

Obs	Parm	Estimate	Stderr	LowerCL	UpperCL	Z	ProbZ
1	Intercept	-7.0656	0.6224	-8.2855	-5.8458	-11.35	<.0001
2	IgG_sero	-0.7011	0.2739	-1.2379	-0.1643	-2.56	0.0105
3	Age	0.0623	0.0050	0.0524	0.0721	12.38	<.0001
4	antibacterial_ever	1.2162	0.1690	0.8850	1.5474	7.20	<.0001
5	arterial_hypertension	0.3493	0.0962	0.1606	0.5379	3.63	0.0003
6	corticosteroids_ever	0.4724	0.1151	0.2468	0.6980	4.10	<.0001
7	diabetes	-0.7222	0.2317	-1.1764	-0.2680	-3.12	0.0018
8	gender2	-0.3594	0.1290	-0.6122	-0.1065	-2.79	0.0053
9	immuno_status	1.1440	0.5372	0.0911	2.1968	2.13	0.0332

Adjusted GEE model for Hospital mortality with IgM_sero

Obs	Parm	Estimate	Stderr	LowerCL	UpperCL	Z	ProbZ
1	Intercept	-6.5573	0.6677	-7.8660	-5.2487	-9.82	<.0001
2	IgM_sero	-0.8929	0.1598	-1.2061	-0.5796	-5.59	<.0001
3	Age	0.0553	0.0059	0.0437	0.0669	9.35	<.0001
4	antibacterial_ever	1.2642	0.1849	0.9018	1.6266	6.84	<.0001
5	arterial_hypertension	0.5916	0.1348	0.3274	0.8559	4.39	<.0001
6	corticosteroids_ever	0.4933	0.1309	0.2367	0.7498	3.77	0.0002
7	diabetes	-0.9789	0.3057	-1.5780	-0.3798	-3.20	0.0014
8	immuno_status	0.7353	0.3598	0.0301	1.4404	2.04	0.0410
9	other_therapy_ever	0.1910	0.0746	0.0449	0.3371	2.56	0.0104

Adjusted GEE model for Hospital mortality with log10IgG

Obs	Parm	Estimate	Stderr	LowerCL	UpperCL	Z	ProbZ
1	Intercept	-6.8880	0.6407	-8.1438	-5.6321	-10.75	<.0001
2	IgG_NIBSC_avg	-0.5659	0.1156	-0.7925	-0.3394	-4.90	<.0001
3	Age	0.0611	0.0055	0.0503	0.0720	11.03	<.0001
4	antibacterial_ever	1.3083	0.2103	0.8960	1.7205	6.22	<.0001
5	arterial_hypertension	0.3955	0.1113	0.1773	0.6137	3.55	0.0004
6	corticosteroids_ever	0.4402	0.0726	0.2979	0.5825	6.06	<.0001
7	diabetes	-0.7952	0.2615	-1.3078	-0.2827	-3.04	0.0024
8	gender2	-0.3707	0.1274	-0.6205	-0.1210	-2.91	0.0036
9	immuno_status	1.1865	0.5652	0.0787	2.2944	2.10	0.0358

Adjusted GEE model for Hospital mortality with log10IgM

Obs	Parm	Estimate	Stderr	LowerCL	UpperCL	Z	ProbZ
**1**	Intercept	-6.5064	0.7152	-7.9081	-5.1047	-9.10	<.0001
**2**	IgM_NIBSC_avg	-0.3122	0.0403	-0.3912	-0.2333	-7.75	<.0001
**3**	Age	0.0546	0.0064	0.0420	0.0673	8.49	<.0001
**4**	antibacterial_ever	1.2800	0.2044	0.8793	1.6806	6.26	<.0001
**5**	arterial_hypertension	0.5533	0.1236	0.3110	0.7956	4.48	<.0001
**6**	corticosteroids_ever	0.4273	0.1488	0.1357	0.7189	2.87	0.0041
**7**	diabetes	-0.9376	0.3203	-1.5652	-0.3099	-2.93	0.0034
**8**	immuno_status	0.8238	0.3703	0.0981	1.5495	2.22	0.0261
**9**	other_therapy_ever	0.2048	0.0977	0.0132	0.3964	2.10	0.0361
