# Supplementary material for: Comprehensive antibody and cytokine profiling in hospitalized COVID-19 patients in relation to clinical outcomes in a large Belgian cohort
Source: Sci Rep. 2023 Nov 7;13:19322. doi: 10.1038/s41598-023-46421-4 (PMC10630327; doi:10.1038/s41598-023-46421-4)
Supplement: Supplementary file 1 — Supplementary Information. [file 41598_2023_46421_MOESM1_ESM.zip › Adjusted GEE model for hospital mortality with CYT.pdf]

Adjusted GEE model for hospital_mortality with log10IFNl1

Obs	Parm	Estimate	Stderr	LowerCL	UpperCL	Z	ProbZ
1	Intercept	-11.3772	1.2638	-13.8542	-8.9002	-9.00	<.0001
2	log10IFNL1	2.0352	0.7913	0.4843	3.5860	2.57	0.0101
3	Age	0.0661	0.0051	0.0561	0.0762	12.88	<.0001
4	antibacterial_ever	1.0701	0.3032	0.4758	1.6643	3.53	0.0004
5	kidney_injury	1.1662	0.3724	0.4363	1.8961	3.13	0.0017
6	other_therapy_ever	0.7153	0.2161	0.2917	1.1388	3.31	0.0009

Adjusted GEE model for hospital_mortality with log10IFNa

Obs	Parm	Estimate	Stderr	LowerCL	UpperCL	Z	ProbZ
1	Intercept	-2.0715	0.4441	-2.9420	-1.2010	-4.66	<.0001
2	log10IFNa	0.7062	0.2877	0.1423	1.2702	2.45	0.0141

Adjusted GEE model for hospital_mortality with log10IFNb

Obs	Parm	Estimate	Stderr	LowerCL	UpperCL	Z	ProbZ
1	Intercept	-9.1280	0.5647	-10.2348	-8.0212	-16.16	<.0001
2	log10IFNb	1.1522	0.3381	0.4895	1.8150	3.41	0.0007
3	Age	0.0588	0.0084	0.0423	0.0753	6.98	<.0001
4	antibacterial_ever	0.9242	0.2320	0.4695	1.3789	3.98	<.0001
5	arterial_hypertension	0.5042	0.2296	0.0542	0.9541	2.20	0.0281
6	kidney_injury	0.8804	0.3973	0.1018	1.6590	2.22	0.0267
7	other_therapy_ever	0.4983	0.1620	0.1808	0.8157	3.08	0.0021

Adjusted GEE model for hospital_mortality with log10IFNg

Obs	Parm	Estimate	Stderr	LowerCL	UpperCL	Z	ProbZ
1	Intercept	-8.6525	0.2779	-9.1973	-8.1078	-31.13	<.0001
2	log10IFNg	0.5460	0.0619	0.4247	0.6673	8.82	<.0001
3	Age	0.0645	0.0060	0.0527	0.0764	10.69	<.0001
4	antibacterial_ever	1.2168	0.2936	0.6413	1.7922	4.14	<.0001
5	kidney_injury	0.8923	0.2713	0.3605	1.4240	3.29	0.0010
6	lung_disease	1.2857	0.6524	0.0069	2.5644	1.97	0.0488
7	other_therapy_ever	0.5310	0.1777	0.1827	0.8792	2.99	0.0028

Adjusted GEE model for hospital_mortality with log10IFNl23

Obs	Parm	Estimate	Stderr	LowerCL	UpperCL	Z	ProbZ
1	Intercept	-6.3145	0.4808	-7.2568	-5.3722	-13.13	<.0001
2	log10IFNl23	-0.3462	0.1589	-0.6577	-0.0348	-2.18	0.0294
3	Age	0.0540	0.0061	0.0421	0.0660	8.86	<.0001
4	antibacterial_ever	1.3399	0.2597	0.8309	1.8489	5.16	<.0001
5	arterial_hypertension	0.5323	0.1824	0.1749	0.8898	2.92	0.0035
6	lung_disease	1.4995	0.6482	0.2291	2.7700	2.31	0.0207

Adjusted GEE model for hospital_mortality with log10IL10

Obs	Parm	Estimate	Stderr	LowerCL	UpperCL	Z	ProbZ
1	Intercept	-13.8523	1.1981	-16.2006	-11.5039	-11.56	<.0001
2	log10IL10	3.0950	0.5326	2.0512	4.1388	5.81	<.0001
3	Age	0.0895	0.0065	0.0767	0.1023	13.69	<.0001
4	antibacterial_ever	0.6834	0.1762	0.3381	1.0286	3.88	0.0001
5	gender2	-0.2496	0.0935	-0.4328	-0.0664	-2.67	0.0076
6	lung_disease	1.2276	0.3231	0.5944	1.8608	3.80	0.0001
7	other_therapy_ever	0.9850	0.0789	0.8304	1.1396	12.49	<.0001

Adjusted GEE model for hospital_mortality with log10IL12

Obs	Parm	Estimate	Stderr	LowerCL	UpperCL	Z	ProbZ
1	Intercept	-7.8186	0.5543	-8.9049	-6.7322	-14.11	<.0001
2	log10IL12	0.7344	0.5055	-0.2563	1.7252	1.45	0.1462
3	Age	0.0579	0.0087	0.0410	0.0749	6.69	<.0001
4	antibacterial_ever	1.2822	0.3311	0.6333	1.9311	3.87	0.0001
5	arterial_hypertension	0.6235	0.2908	0.0535	1.1935	2.14	0.0320
6	kidney_injury	0.8910	0.2932	0.3164	1.4656	3.04	0.0024
7	lung_disease	1.2569	0.5999	0.0811	2.4327	2.10	0.0362
8	other_therapy_ever	0.4577	0.1449	0.1738	0.7417	3.16	0.0016

Adjusted GEE model for hospital_mortality with log10IL6

Obs	Parm	Estimate	Stderr	LowerCL	UpperCL	Z	ProbZ
1	Intercept	-15.5508	0.3788	-16.2931	-14.8084	-41.06	<.0001
2	log10IL6	3.1559	0.3114	2.5456	3.7662	10.13	<.0001
3	Age	0.0921	0.0082	0.0760	0.1081	11.27	<.0001
4	kidney_injury	0.7556	0.2860	0.1951	1.3161	2.64	0.0082
5	lung_disease	1.2925	0.4740	0.3635	2.2215	2.73	0.0064
6	other_therapy_ever	0.9142	0.1151	0.6886	1.1398	7.94	<.0001

Adjusted GEE model for hospital_mortality with log10IL8

Obs	Parm	Estimate	Stderr	LowerCL	UpperCL	Z	ProbZ
1	Intercept	-12.0888	2.1471	-16.2970	-7.8806	-5.63	<.0001
2	log10IL8	2.2972	0.8333	0.6639	3.9304	2.76	0.0058
3	Age	0.0558	0.0047	0.0466	0.0649	11.96	<.0001
4	antibacterial_ever	1.3135	0.2572	0.8094	1.8177	5.11	<.0001
5	arterial_hypertension	0.7706	0.3036	0.1755	1.3657	2.54	0.0111
6	kidney_injury	0.6830	0.2368	0.2188	1.1472	2.88	0.0039
7	lung_disease	1.2479	0.5487	0.1725	2.3234	2.27	0.0229

Adjusted GEE model for hospital_mortality with log10IP10

Obs	Parm	Estimate	Stderr	LowerCL	UpperCL	Z	ProbZ
1	Intercept	-12.8275	0.5507	-13.9070	-11.7481	-23.29	<.0001
2	log10IP10	2.1997	0.2919	1.6275	2.7719	7.53	<.0001
3	Age	0.0630	0.0052	0.0528	0.0732	12.10	<.0001
4	antibacterial_ever	0.7040	0.3317	0.0539	1.3542	2.12	0.0338
5	hydroxychloroquine_ever	-0.5488	0.2100	-0.9605	-0.1372	-2.61	0.0090
6	other_therapy_ever	0.6550	0.3016	0.0639	1.2460	2.17	0.0299

Adjusted GEE model for hospital_mortality   with log10GM

Obs	Parm	Estimate	Stderr	LowerCL	UpperCL	Z	ProbZ
1	Intercept	-6.9035	0.8266	-8.5236	-5.2835	-8.35	<.0001
2	log10GM	0.0315	0.4412	-0.8333	0.8962	0.07	0.9432
3	Age	0.0530	0.0092	0.0350	0.0710	5.76	<.0001
4	antibacterial_ever	1.3987	0.2470	0.9146	1.8828	5.66	<.0001
5	arterial_hypertension	0.5596	0.2109	0.1463	0.9729	2.65	0.0080
6	lung_disease	1.4670	0.7081	0.0792	2.8549	2.07	0.0383
