# Supplementary material for: Comprehensive antibody and cytokine profiling in hospitalized COVID-19 patients in relation to clinical outcomes in a large Belgian cohort
Source: Sci Rep. 2023 Nov 7;13:19322. doi: 10.1038/s41598-023-46421-4 (PMC10630327; doi:10.1038/s41598-023-46421-4)
Supplement: Supplementary file 1 — Supplementary Information. [file 41598_2023_46421_MOESM1_ESM.zip › Adjusted GEE model for ICU admission with AB.pdf]

Adjusted GEE model for ICU_admission with IgG_sero

Obs	Parm	Estimate	Stderr	LowerCL	UpperCL	Z	ProbZ
1	Intercept	-0.7110	0.3164	-1.3312	-0.0908	-2.25	0.0246
2	IgG_sero	0.3052	0.2528	-0.1903	0.8007	1.21	0.2273
3	Age	-0.0308	0.0035	-0.0377	-0.0239	-8.77	<.0001
4	corticosteroids_ever	1.0412	0.4702	0.1196	1.9628	2.21	0.0268
5	gender2	-0.6925	0.0772	-0.8437	-0.5413	-8.98	<.0001
6	hydroxychloroquine_ever	1.7491	0.7715	0.2370	3.2612	2.27	0.0234
7	kidney_injury	1.7258	0.1836	1.3660	2.0857	9.40	<.0001

Adjusted GEE model for ICU_admission with IgM_sero

Obs	Parm	Estimate	Stderr	LowerCL	UpperCL	Z	ProbZ
1	Intercept	-0.3369	0.3119	-0.9483	0.2745	-1.08	0.2801
2	IgM_sero	-0.3581	0.1429	-0.6382	-0.0779	-2.50	0.0123
3	Age	-0.0320	0.0035	-0.0389	-0.0252	-9.19	<.0001
4	corticosteroids_ever	1.0534	0.4709	0.1305	1.9763	2.24	0.0253
5	gender2	-0.7704	0.0961	-0.9588	-0.5821	-8.02	<.0001
6	hydroxychloroquine_ever	1.7503	0.7787	0.2240	3.2765	2.25	0.0246
7	kidney_injury	1.5442	0.2564	1.0416	2.0467	6.02	<.0001

Adjusted GEE model for ICU_admission with log10IgG

Obs	Parm	Estimate	Stderr	LowerCL	UpperCL	Z	ProbZ
1	Intercept	-0.6703	0.3513	-1.3589	0.0183	-1.91	0.0564
2	IgG_NIBSC_avg	0.0711	0.1389	-0.2012	0.3433	0.51	0.6089
3	Age	-0.0309	0.0036	-0.0379	-0.0239	-8.64	<.0001
4	corticosteroids_ever	1.0403	0.4645	0.1300	1.9507	2.24	0.0251
5	gender2	-0.6879	0.0885	-0.8614	-0.5143	-7.77	<.0001
6	hydroxychloroquine_ever	1.7444	0.7702	0.2347	3.2540	2.26	0.0235
7	kidney_injury	1.6921	0.2020	1.2961	2.0880	8.38	<.0001

Adjusted GEE model for ICU_admission with log10IgM

Obs	Parm	Estimate	Stderr	LowerCL	UpperCL	Z	ProbZ
1	Intercept	-0.5156	0.3291	-1.1605	0.1294	-1.57	0.1172
2	IgM_NIBSC_avg	-0.0459	0.0664	-0.1761	0.0842	-0.69	0.4893
3	Age	-0.0306	0.0034	-0.0373	-0.0240	-9.04	<.0001
4	corticosteroids_ever	1.0694	0.4656	0.1568	1.9820	2.30	0.0216
5	gender2	-0.7667	0.1042	-0.9709	-0.5625	-7.36	<.0001
6	hydroxychloroquine_ever	1.6868	0.7891	0.1401	3.2334	2.14	0.0326
7	kidney_injury	1.5235	0.2483	1.0368	2.0102	6.14	<.0001
