# Supplementary material for: Comprehensive antibody and cytokine profiling in hospitalized COVID-19 patients in relation to clinical outcomes in a large Belgian cohort
Source: Sci Rep. 2023 Nov 7;13:19322. doi: 10.1038/s41598-023-46421-4 (PMC10630327; doi:10.1038/s41598-023-46421-4)
Supplement: Supplementary file 1 — Supplementary Information. [file 41598_2023_46421_MOESM1_ESM.zip › Adjusted GEE model for ICU mortality with AB.pdf]

Adjusted GEE model for ICU_mortality with IgG_sero

Obs	Parm	Estimate	Stderr	LowerCL	UpperCL	Z	ProbZ
1	Intercept	-31.0798	4.2461	-39.4021	-22.7575	-7.32	<.0001
2	IgG_sero	-2.9620	1.4750	-5.8530	-0.0709	-2.01	0.0446
3	Age	0.3144	0.0265	0.2625	0.3663	11.87	<.0001
4	BMI_total	0.1747	0.0878	0.0027	0.3468	1.99	0.0465
5	antibacterial_ever	3.4809	1.4668	0.6060	6.3558	2.37	0.0176
6	corticosteroids_ever	-1.7376	0.8355	-3.3751	-0.1002	-2.08	0.0375
7	diabetes	-5.2739	1.7932	-8.7885	-1.7593	-2.94	0.0033
8	gender2	2.8450	0.0930	2.6627	3.0272	30.59	<.0001
9	hydroxychloroquine_ever	-4.2509	1.2159	-6.6340	-1.8678	-3.50	0.0005
10	immuno_status	4.8403	1.0676	2.7478	6.9328	4.53	<.0001

Adjusted GEE model for ICU_mortality with IgM_sero

Obs	Parm	Estimate	Stderr	LowerCL	UpperCL	Z	ProbZ
1	Intercept	-7.7329	0.8945	-9.4861	-5.9797	-8.64	<.0001
2	IgM_sero	-0.5130	0.3490	-1.1969	0.1709	-1.47	0.1415
3	Age	0.1013	0.0178	0.0665	0.1362	5.71	<.0001
4	diabetes	-1.7709	0.7066	-3.1559	-0.3859	-2.51	0.0122

Adjusted GEE model for ICU_mortality with log10IgG

Obs	Parm	Estimate	Stderr	LowerCL	UpperCL	Z	ProbZ
1	Intercept	-10.6971	2.2134	-15.0352	-6.3589	-4.83	<.0001
2	IgG_NIBSC_avg	-0.3689	0.5664	-1.4790	0.7412	-0.65	0.5148
3	Age	0.1270	0.0339	0.0606	0.1935	3.75	0.0002
4	arterial_hypertension	1.5018	0.7021	0.1258	2.8779	2.14	0.0324
5	diabetes	-2.4331	0.9036	-4.2041	-0.6621	-2.69	0.0071
6	gender2	0.6562	0.2182	0.2286	1.0839	3.01	0.0026

Adjusted GEE model for ICU_mortality with log10IgM

Obs	Parm	Estimate	Stderr	LowerCL	UpperCL	Z	ProbZ
1	Intercept	-9.2520	1.5235	-12.2381	-6.2659	-6.07	<.0001
2	IgM_NIBSC_avg	-0.6376	0.2274	-1.0833	-0.1920	-2.80	0.0050
3	Age	0.1034	0.0232	0.0581	0.1488	4.47	<.0001
4	antibacterial_ever	2.2548	0.9034	0.4842	4.0254	2.50	0.0126
5	corticosteroids_ever	-1.4967	0.3904	-2.2619	-0.7316	-3.83	0.0001
6	diabetes	-3.5024	0.9804	-5.4239	-1.5808	-3.57	0.0004
7	hydroxychloroquine_ever	-1.7700	0.6368	-3.0181	-0.5219	-2.78	0.0054
8	immuno_status	3.4289	1.0781	1.3158	5.5420	3.18	0.0015
9	kidney_injury	2.4302	0.5079	1.4348	3.4257	4.79	<.0001
