# Supplementary material for: Comprehensive antibody and cytokine profiling in hospitalized COVID-19 patients in relation to clinical outcomes in a large Belgian cohort
Source: Sci Rep. 2023 Nov 7;13:19322. doi: 10.1038/s41598-023-46421-4 (PMC10630327; doi:10.1038/s41598-023-46421-4)
Supplement: Supplementary file 1 — Supplementary Information. [file 41598_2023_46421_MOESM1_ESM.zip › Adjusted GEE model for Ln(CRP) with AB.pdf]

Adjusted GEE model for lncrp with IgG_sero

Obs	Parm	Estimate	Stderr	LowerCL	UpperCL	Z	ProbZ
1	Intercept	2.3241	0.3510	1.6363	3.0120	6.62	<.0001
2	IgG_sero	0.3356	0.0945	0.1505	0.5207	3.55	0.0004
3	Age	0.0069	0.0026	0.0018	0.0120	2.64	0.0082
4	antibacterial_ever	1.2662	0.2507	0.7749	1.7575	5.05	<.0001
5	gender2	-0.4447	0.0579	-0.5582	-0.3312	-7.68	<.0001
6	hydroxychloroquine_ever	0.5908	0.0444	0.5038	0.6777	13.31	<.0001

Adjusted GEE model for lncrp with IgM_sero

Obs	Parm	Estimate	Stderr	LowerCL	UpperCL	Z	ProbZ
1	Intercept	1.8847	0.3380	1.2223	2.5471	5.58	<.0001
2	IgM_sero	0.5932	0.1089	0.3798	0.8066	5.45	<.0001
3	Age	0.0101	0.0024	0.0053	0.0149	4.13	<.0001
4	antibacterial_ever	1.2332	0.1800	0.8803	1.5860	6.85	<.0001
5	gender2	-0.4367	0.0570	-0.5484	-0.3250	-7.66	<.0001
6	hydroxychloroquine_ever	0.4778	0.0514	0.3771	0.5784	9.30	<.0001
7	malignancies	0.2501	0.1258	0.0035	0.4967	1.99	0.0469

Adjusted GEE model for lncrp with log10IgG

Obs	Parm	Estimate	Stderr	LowerCL	UpperCL	Z	ProbZ
1	Intercept	2.2395	0.3536	1.5465	2.9325	6.33	<.0001
2	IgG_NIBSC_avg	0.1744	0.0493	0.0777	0.2711	3.53	0.0004
3	Age	0.0075	0.0027	0.0022	0.0127	2.78	0.0055
4	antibacterial_ever	1.3163	0.2510	0.8244	1.8082	5.24	<.0001
5	gender2	-0.4559	0.0615	-0.5765	-0.3352	-7.41	<.0001
6	hydroxychloroquine_ever	0.5589	0.0244	0.5111	0.6066	22.94	<.0001
7	lung_disease	-0.2324	0.1182	-0.4640	-0.0007	-1.97	0.0493

Adjusted GEE model for lncrp with log10IgM

Obs	Parm	Estimate	Stderr	LowerCL	UpperCL	Z	ProbZ
1	Intercept	1.9182	0.3427	1.2465	2.5899	5.60	<.0001
2	IgM_NIBSC_avg	0.1846	0.0262	0.1332	0.2360	7.04	<.0001
3	Age	0.0095	0.0025	0.0045	0.0144	3.75	0.0002
4	antibacterial_ever	1.2492	0.1901	0.8765	1.6219	6.57	<.0001
5	gender2	-0.3965	0.0456	-0.4858	-0.3072	-8.70	<.0001
6	hydroxychloroquine_ever	0.4971	0.0384	0.4217	0.5724	12.94	<.0001
7	malignancies	0.2289	0.1113	0.0108	0.4471	2.06	0.0397
