# Supplementary material for: Comprehensive antibody and cytokine profiling in hospitalized COVID-19 patients in relation to clinical outcomes in a large Belgian cohort
Source: Sci Rep. 2023 Nov 7;13:19322. doi: 10.1038/s41598-023-46421-4 (PMC10630327; doi:10.1038/s41598-023-46421-4)
Supplement: Supplementary file 1 — Supplementary Information. [file 41598_2023_46421_MOESM1_ESM.zip › Adjusted GEE model for Ln(CRP) with CYT.pdf]

Adjusted GEE model for lncrp with log10IFNl1

Obs	Parm	Estimate	Stderr	LowerCL	UpperCL	Z	ProbZ
1	Intercept	1.6944	0.5678	0.5816	2.8072	2.98	0.0028
2	log10IFNL1	0.4639	0.1228	0.2231	0.7046	3.78	0.0002
3	Age	0.0096	0.0040	0.0017	0.0174	2.39	0.0167
4	antibacterial_ever	1.0426	0.1713	0.7069	1.3783	6.09	<.0001
5	diabetes	0.3314	0.1188	0.0986	0.5642	2.79	0.0053
6	gender2	-0.4170	0.1209	-0.6540	-0.1800	-3.45	0.0006
7	hydroxychloroquine_ever	0.5769	0.0599	0.4595	0.6943	9.63	<.0001
8	other_therapy_ever	-0.1557	0.0497	-0.2531	-0.0583	-3.13	0.0017

Adjusted GEE model for lncrp with log10IFNa

Obs	Parm	Estimate	Stderr	LowerCL	UpperCL	Z	ProbZ
1	Intercept	2.3432	0.4300	1.5003	3.1861	5.45	<.0001
2	log10IFNa	0.1514	0.0732	0.0080	0.2948	2.07	0.0385
3	Age	0.0102	0.0046	0.0011	0.0193	2.20	0.0279
4	antibacterial_ever	1.0315	0.1776	0.6834	1.3796	5.81	<.0001
5	diabetes	0.3108	0.1279	0.0601	0.5614	2.43	0.0151
6	gender2	-0.4106	0.1249	-0.6554	-0.1658	-3.29	0.0010
7	hydroxychloroquine_ever	0.5931	0.0668	0.4622	0.7239	8.88	<.0001
8	other_therapy_ever	-0.2357	0.0558	-0.3451	-0.1262	-4.22	<.0001

Adjusted GEE model for lncrp with log10IFNb

Obs	Parm	Estimate	Stderr	LowerCL	UpperCL	Z	ProbZ
1	Intercept	1.5643	0.4872	0.6094	2.5193	3.21	0.0013
2	log10IFNb	0.5030	0.0611	0.3832	0.6227	8.23	<.0001
3	Age	0.0099	0.0048	0.0006	0.0192	2.08	0.0377
4	antibacterial_ever	1.0805	0.1877	0.7126	1.4483	5.76	<.0001
5	gender2	-0.4738	0.0905	-0.6511	-0.2965	-5.24	<.0001
6	hydroxychloroquine_ever	0.5357	0.0783	0.3823	0.6891	6.84	<.0001

Adjusted GEE model for lncrp with log10IFNg

Obs	Parm	Estimate	Stderr	LowerCL	UpperCL	Z	ProbZ
1	Intercept	1.6033	0.5731	0.4801	2.7265	2.80	0.0051
2	log10IFNg	0.3922	0.0758	0.2436	0.5407	5.17	<.0001
3	Age	0.0122	0.0049	0.0025	0.0219	2.47	0.0136
4	antibacterial_ever	1.0927	0.1743	0.7511	1.4344	6.27	<.0001
5	gender2	-0.4214	0.1051	-0.6274	-0.2154	-4.01	<.0001
6	hydroxychloroquine_ever	0.5142	0.0677	0.3815	0.6469	7.60	<.0001

Adjusted GEE model for lncrp with log10IFNl23

Obs	Parm	Estimate	Stderr	LowerCL	UpperCL	Z	ProbZ
1	Intercept	3.0616	0.2952	2.4830	3.6402	10.37	<.0001
2	log10IFNl23	0.0403	0.0962	-0.1482	0.2287	0.42	0.6754
3	antibacterial_ever	1.0527	0.1981	0.6644	1.4410	5.31	<.0001
4	diabetes	0.3415	0.1446	0.0581	0.6249	2.36	0.0182
5	gender2	-0.3735	0.1389	-0.6457	-0.1013	-2.69	0.0072
6	hydroxychloroquine_ever	0.5891	0.0704	0.4510	0.7272	8.36	<.0001
7	kidney_injury	0.4196	0.1618	0.1025	0.7367	2.59	0.0095
8	other_therapy_ever	-0.2235	0.0614	-0.3438	-0.1032	-3.64	0.0003

Adjusted GEE model for lncrp with log10IL10

Obs	Parm	Estimate	Stderr	LowerCL	UpperCL	Z	ProbZ
1	Intercept	1.2458	0.4075	0.4470	2.0445	3.06	0.0022
2	log10IL10	1.0144	0.1005	0.8175	1.2113	10.10	<.0001
3	Age	0.0091	0.0034	0.0023	0.0158	2.64	0.0083
4	antibacterial_ever	0.9130	0.1750	0.5700	1.2560	5.22	<.0001
5	gender2	-0.3752	0.0880	-0.5476	-0.2028	-4.27	<.0001
6	hydroxychloroquine_ever	0.4914	0.0794	0.3356	0.6471	6.19	<.0001
7	immuno_status	-0.5096	0.2459	-0.9915	-0.0277	-2.07	0.0382
8	malignancies	0.2514	0.0558	0.1420	0.3609	4.50	<.0001

Adjusted GEE model for lncrp with log10IL12

Obs	Parm	Estimate	Stderr	LowerCL	UpperCL	Z	ProbZ
1	Intercept	3.1382	0.1488	2.8466	3.4298	21.09	<.0001
2	log10IL12	0.0105	0.1728	-0.3282	0.3492	0.06	0.9516
3	antibacterial_ever	1.0463	0.1827	0.6882	1.4044	5.73	<.0001
4	diabetes	0.3476	0.1394	0.0744	0.6209	2.49	0.0127
5	gender2	-0.3683	0.1407	-0.6441	-0.0926	-2.62	0.0089
6	hydroxychloroquine_ever	0.5891	0.0731	0.4458	0.7325	8.05	<.0001
7	kidney_injury	0.4175	0.1635	0.0971	0.7379	2.55	0.0106
8	other_therapy_ever	-0.2353	0.0685	-0.3697	-0.1010	-3.43	0.0006

Adjusted GEE model for lncrp with log10IL6

Obs	Parm	Estimate	Stderr	LowerCL	UpperCL	Z	ProbZ
1	Intercept	0.8491	0.0965	0.6600	1.0381	8.80	<.0001
2	log10IL6	1.4526	0.0893	1.2775	1.6277	16.26	<.0001
3	antibacterial_ever	0.4864	0.1579	0.1770	0.7958	3.08	0.0021
4	gender2	-0.2615	0.0694	-0.3974	-0.1256	-3.77	0.0002
5	hydroxychloroquine_ever	0.4533	0.0280	0.3985	0.5081	16.20	<.0001
6	immuno_status	-0.3929	0.1612	-0.7088	-0.0770	-2.44	0.0148

Adjusted GEE model for lncrp with log10IL8

Obs	Parm	Estimate	Stderr	LowerCL	UpperCL	Z	ProbZ
1	Intercept	2.1710	0.9182	0.3713	3.9707	2.36	0.0181
2	log10IL8	0.5062	0.4478	-0.3715	1.3838	1.13	0.2583
3	antibacterial_ever	0.9350	0.2129	0.5178	1.3523	4.39	<.0001
4	diabetes	0.3798	0.1357	0.1138	0.6458	2.80	0.0051
5	gender2	-0.3685	0.1256	-0.6148	-0.1223	-2.93	0.0034
6	hydroxychloroquine_ever	0.6834	0.0990	0.4894	0.8774	6.90	<.0001
7	kidney_injury	0.4006	0.1225	0.1604	0.6407	3.27	0.0011
8	other_therapy_ever	-0.2905	0.0923	-0.4714	-0.1096	-3.15	0.0016

Adjusted GEE model for lncrp with log10IP10

Obs	Parm	Estimate	Stderr	LowerCL	UpperCL	Z	ProbZ
1	Intercept	-0.8618	0.3615	-1.5703	-0.1532	-2.38	0.0171
2	log10IP10	1.4071	0.1827	1.0491	1.7652	7.70	<.0001
3	Age	0.0085	0.0021	0.0043	0.0126	4.00	<.0001
4	antibacterial_ever	0.8477	0.1745	0.5058	1.1897	4.86	<.0001
5	arterial_hypertension	-0.1865	0.0512	-0.2869	-0.0861	-3.64	0.0003
6	gender2	-0.3344	0.1259	-0.5811	-0.0878	-2.66	0.0079
7	hydroxychloroquine_ever	0.2114	0.0642	0.0855	0.3372	3.29	0.0010

Adjusted GEE model for lncrp with log10GM

Obs	Parm	Estimate	Stderr	LowerCL	UpperCL	Z	ProbZ
1	Intercept	3.0304	0.1958	2.6467	3.4142	15.48	<.0001
2	log10GM	0.1348	0.2536	-0.3623	0.6319	0.53	0.5951
3	antibacterial_ever	1.0439	0.1889	0.6736	1.4142	5.53	<.0001
4	diabetes	0.3584	0.1168	0.1294	0.5874	3.07	0.0022
5	gender2	-0.3689	0.1356	-0.6346	-0.1031	-2.72	0.0065
6	hydroxychloroquine_ever	0.5776	0.0743	0.4319	0.7233	7.77	<.0001
7	kidney_injury	0.4159	0.1709	0.0808	0.7509	2.43	0.0150
8	other_therapy_ever	-0.2368	0.0720	-0.3780	-0.0957	-3.29	0.0010
