# Supplementary material for: Comprehensive antibody and cytokine profiling in hospitalized COVID-19 patients in relation to clinical outcomes in a large Belgian cohort
Source: Sci Rep. 2023 Nov 7;13:19322. doi: 10.1038/s41598-023-46421-4 (PMC10630327; doi:10.1038/s41598-023-46421-4)
Supplement: Supplementary file 1 — Supplementary Information. [file 41598_2023_46421_MOESM1_ESM.zip › Adjusted GEE model for Ln(D-dimers) with AB.pdf]

Adjusted GEE model for lnd_dimeren with IgG_sero

Obs	Parm	Estimate	Stderr	LowerCL	UpperCL	Z	ProbZ
1	Intercept	-1.0591	0.0837	-1.2231	-0.8951	-12.66	<.0001
2	IgG_sero	0.5783	0.1788	0.2278	0.9287	3.23	0.0012
3	Age	0.0134	0.0017	0.0101	0.0166	8.03	<.0001
4	antibacterial_ever	0.1499	0.0349	0.0814	0.2184	4.29	<.0001

Adjusted GEE model for lnd_dimeren with IgM_sero

Obs	Parm	Estimate	Stderr	LowerCL	UpperCL	Z	ProbZ
1	Intercept	-1.2169	0.1265	-1.4649	-0.9688	-9.62	<.0001
2	IgM_sero	0.3893	0.0965	0.2002	0.5785	4.03	<.0001
3	Age	0.0149	0.0025	0.0100	0.0198	5.99	<.0001
4	antibacterial_ever	0.1037	0.0274	0.0500	0.1574	3.78	0.0002

Adjusted GEE model for lnd_dimeren with log10IgG

Obs	Parm	Estimate	Stderr	LowerCL	UpperCL	Z	ProbZ
1	Intercept	-1.0721	0.1137	-1.2949	-0.8493	-9.43	<.0001
2	IgG_NIBSC_avg	0.4103	0.0798	0.2539	0.5667	5.14	<.0001
3	Age	0.0127	0.0016	0.0095	0.0158	7.81	<.0001
4	corticosteroids_ever	0.3063	0.1241	0.0631	0.5496	2.47	0.0136

Adjusted GEE model for lnd_dimeren with log10IgM

Obs	Parm	Estimate	Stderr	LowerCL	UpperCL	Z	ProbZ
1	Intercept	-1.7250	0.0741	-1.8702	-1.5798	-23.29	<.0001
2	IgM_NIBSC_avg	0.1677	0.0268	0.1152	0.2201	6.27	<.0001
3	Age	0.0228	0.0011	0.0207	0.0249	20.92	<.0001
4	antibacterial_ever	0.1386	0.0449	0.0506	0.2267	3.09	0.0020
5	arterial_hypertension	-0.1758	0.0630	-0.2993	-0.0523	-2.79	0.0053
6	diabetes	0.2721	0.0217	0.2295	0.3147	12.52	<.0001
7	other_therapy_ever	-0.2120	0.0935	-0.3953	-0.0287	-2.27	0.0234
