# Supplementary material for: Comprehensive antibody and cytokine profiling in hospitalized COVID-19 patients in relation to clinical outcomes in a large Belgian cohort
Source: Sci Rep. 2023 Nov 7;13:19322. doi: 10.1038/s41598-023-46421-4 (PMC10630327; doi:10.1038/s41598-023-46421-4)
Supplement: Supplementary file 1 — Supplementary Information. [file 41598_2023_46421_MOESM1_ESM.zip › Adjusted GEE model for Ln(D-dimers) with CYT.pdf]

Adjusted GEE model for lnd_dimeren with log10IFNl1

Obs	Parm	Estimate	Stderr	LowerCL	UpperCL	Z	ProbZ
1	Intercept	-0.4075	0.7706	-1.9179	1.1029	-0.53	0.5969
2	log10IFNL1	-0.3816	0.1542	-0.6838	-0.0795	-2.48	0.0133
3	Age	0.0190	0.0063	0.0067	0.0313	3.03	0.0024
4	antibacterial_ever	0.2911	0.0617	0.1701	0.4121	4.72	<.0001
5	gender2	0.1581	0.0246	0.1100	0.2063	6.43	<.0001
6	other_therapy_ever	-0.5706	0.1425	-0.8499	-0.2913	-4.00	<.0001

Adjusted GEE model for lnd_dimeren with log10IFNa

Obs	Parm	Estimate	Stderr	LowerCL	UpperCL	Z	ProbZ
1	Intercept	0.1971	0.0925	0.0158	0.3783	2.13	0.0331
2	log10IFNa	-0.2544	0.0829	-0.4170	-0.0919	-3.07	0.0022
3	antibacterial_ever	0.2962	0.1095	0.0816	0.5108	2.71	0.0068

Adjusted GEE model for lnd_dimeren with log10IFNb

Obs	Parm	Estimate	Stderr	LowerCL	UpperCL	Z	ProbZ
1	Intercept	0.0256	0.3295	-0.6202	0.6714	0.08	0.9381
2	log10IFNb	-0.0555	0.1632	-0.3754	0.2644	-0.34	0.7337
3	antibacterial_ever	0.3186	0.1341	0.0558	0.5814	2.38	0.0175

Adjusted GEE model for lnd_dimeren with log10IFNg

Obs	Parm	Estimate	Stderr	LowerCL	UpperCL	Z	ProbZ
1	Intercept	-1.3161	0.5244	-2.3439	-0.2883	-2.51	0.0121
2	log10IFNg	0.1514	0.0970	-0.0388	0.3415	1.56	0.1187
3	Age	0.0182	0.0067	0.0050	0.0313	2.71	0.0066
4	antibacterial_ever	0.2729	0.0648	0.1459	0.4000	4.21	<.0001
5	gender2	0.1976	0.0403	0.1187	0.2765	4.91	<.0001
6	other_therapy_ever	-0.4896	0.1064	-0.6980	-0.2811	-4.60	<.0001

Adjusted GEE model for lnd_dimeren with log10IFNl23

Obs	Parm	Estimate	Stderr	LowerCL	UpperCL	Z	ProbZ
1	Intercept	-0.5502	0.2230	-0.9874	-0.1130	-2.47	0.0136
2	log10IFNl23	0.2477	0.1079	0.0363	0.4591	2.30	0.0217
3	antibacterial_ever	0.3579	0.1158	0.1310	0.5849	3.09	0.0020

Adjusted GEE model for lnd_dimeren with log10IL10

Obs	Parm	Estimate	Stderr	LowerCL	UpperCL	Z	ProbZ
1	Intercept	0.0823	0.3247	-0.5542	0.7187	0.25	0.8000
2	log10IL10	0.0975	0.2089	-0.3119	0.5069	0.47	0.6407
3	antibacterial_ever	0.3007	0.0760	0.1519	0.4496	3.96	<.0001
4	gender2	0.2822	0.0697	0.1456	0.4189	4.05	<.0001
5	other_therapy_ever	-0.5501	0.1277	-0.8003	-0.2998	-4.31	<.0001

Adjusted GEE model for Ind_dimeren with log10IL12

Obs	Parm	Estimate	Stderr	LowerCL	UpperCL	Z	ProbZ
1	Intercept	-0.1377	0.1585	-0.4483	0.1729	-0.87	0.3849
2	log10IL12	0.2830	0.2136	-0.1357	0.7016	1.32	0.1852
3	antibacterial_ever	0.3019	0.1213	0.0641	0.5396	2.49	0.0128

Adjusted GEE model for lnd_dimeren with log10IL6

Obs	Parm	Estimate	Stderr	LowerCL	UpperCL	Z	ProbZ
1	Intercept	-0.1395	0.3081	-0.7435	0.4644	-0.45	0.6506
2	log10IL6	0.2138	0.1106	-0.0030	0.4305	1.93	0.0532
3	antibacterial_ever	0.2216	0.0760	0.0726	0.3706	2.91	0.0036
4	gender2	0.2884	0.0742	0.1429	0.4338	3.89	0.0001
5	other_therapy_ever	-0.5406	0.1220	-0.7796	-0.3016	-4.43	<.0001

Adjusted GEE model for lnd_dimeren with log10IL8

Obs	Parm	Estimate	Stderr	LowerCL	UpperCL	Z	ProbZ
1	Intercept	-1.7459	1.0193	-3.7437	0.2518	-1.71	0.0867
2	log10IL8	0.5467	0.2750	0.0077	1.0857	1.99	0.0468
3	Age	0.0155	0.0051	0.0055	0.0256	3.03	0.0025
4	gender2	0.1788	0.0698	0.0420	0.3155	2.56	0.0104
5	other_therapy_ever	-0.5717	0.0859	-0.7402	-0.4033	-6.65	<.0001

Adjusted GEE model for lnd_dimeren with log10IP10

Obs	Parm	Estimate	Stderr	LowerCL	UpperCL	Z	ProbZ
1	Intercept	1.6802	0.9156	-0.1143	3.4748	1.84	0.0665
2	log10IP10	-0.5504	0.2908	-1.1204	0.0195	-1.89	0.0584
3	antibacterial_ever	0.4694	0.1019	0.2698	0.6691	4.61	<.0001
4	gender2	0.1857	0.0777	0.0334	0.3380	2.39	0.0168
5	other_therapy_ever	-0.6196	0.1613	-0.9356	-0.3035	-3.84	0.0001

Adjusted GEE model for lnd_dimeren with log10GM

Obs	Parm	Estimate	Stderr	LowerCL	UpperCL	Z	ProbZ
1	Intercept	-0.1974	0.2078	-0.6047	0.2099	-0.95	0.3422
2	log10GM	0.1492	0.1155	-0.0771	0.3756	1.29	0.1962
3	antibacterial_ever	0.3222	0.1307	0.0660	0.5783	2.47	0.0137
