# Supplementary material for: Comprehensive antibody and cytokine profiling in hospitalized COVID-19 patients in relation to clinical outcomes in a large Belgian cohort
Source: Sci Rep. 2023 Nov 7;13:19322. doi: 10.1038/s41598-023-46421-4 (PMC10630327; doi:10.1038/s41598-023-46421-4)
Supplement: Supplementary file 1 — Supplementary Information. [file 41598_2023_46421_MOESM1_ESM.zip › Adjusted GEE model for Ln(Ferritin) with AB.pdf]

Adjusted GEE model for Inferritine with IgG_sero

Obs	Parm	Estimate	Stderr	LowerCL	UpperCL	Z	ProbZ
1	Intercept	6.3026	0.2882	5.7377	6.8676	21.87	<.0001
2	IgG_sero	0.1311	0.1405	-0.1443	0.4065	0.93	0.3509
3	gender2	-0.8476	0.2192	-1.2772	-0.4180	-3.87	0.0001
4	hydroxychloroquine_ever	0.4819	0.1822	0.1248	0.8390	2.64	0.0082
5	malignancies	0.5807	0.0671	0.4492	0.7122	8.65	<.0001

Adjusted GEE model for lnferritine with IgM_sero

Obs	Parm	Estimate	Stderr	LowerCL	UpperCL	Z	ProbZ
1	Intercept	6.2517	0.2201	5.8203	6.6831	28.40	<.0001
2	IgM_sero	0.4627	0.0997	0.2673	0.6581	4.64	<.0001
3	gender2	-0.7979	0.2148	-1.2188	-0.3769	-3.71	0.0002
4	malignancies	0.7766	0.1235	0.5346	1.0185	6.29	<.0001

Adjusted GEE model for Inferritine with log10IgG

Obs	Parm	Estimate	Stderr	LowerCL	UpperCL	Z	ProbZ
1	Intercept	6.2291	0.2682	5.7033	6.7548	23.22	<.0001
2	IgG_NIBSC_avg	0.1629	0.0499	0.0650	0.2608	3.26	0.0011
3	gender2	-0.8178	0.2127	-1.2347	-0.4010	-3.85	0.0001
4	hydroxychloroquine_ever	0.4522	0.1894	0.0811	0.8234	2.39	0.0169
5	malignancies	0.5745	0.0632	0.4506	0.6985	9.09	<.0001

Adjusted GEE model for lnferritine with log10IgM

Obs	Parm	Estimate	Stderr	LowerCL	UpperCL	Z	ProbZ
1	Intercept	6.1777	0.2171	5.7523	6.6031	28.46	<.0001
2	IgM_NIBSC_avg	0.1849	0.0371	0.1122	0.2577	4.98	<.0001
3	gender2	-0.7382	0.2052	-1.1404	-0.3361	-3.60	0.0003
4	malignancies	0.7647	0.1529	0.4651	1.0644	5.00	<.0001
