# Supplementary material for: Comprehensive antibody and cytokine profiling in hospitalized COVID-19 patients in relation to clinical outcomes in a large Belgian cohort
Source: Sci Rep. 2023 Nov 7;13:19322. doi: 10.1038/s41598-023-46421-4 (PMC10630327; doi:10.1038/s41598-023-46421-4)
Supplement: Supplementary file 1 — Supplementary Information. [file 41598_2023_46421_MOESM1_ESM.zip › Adjusted GEE model for Ln(ferritin) with CYT.pdf]

Adjusted GEE model lnferritine with log10IFNl1

Obs	Parm	Estimate	Stderr	LowerCL	UpperCL	Z	ProbZ
1	Intercept	5.1773	0.7025	3.8005	6.5541	7.37	<.0001
2	log10IFNL1	1.4748	0.1683	1.1449	1.8047	8.76	<.0001
3	BMI_total	-0.0508	0.0203	-0.0906	-0.0111	-2.51	0.0121
4	lung_disease	-2.9552	0.2960	-3.5355	-2.3750	-9.98	<.0001

Adjusted GEE model lnferritine   with log10IFNa

Obs	Parm	Estimate	Stderr	LowerCL	UpperCL	Z	ProbZ
1	Intercept	7.2858	0.5493	6.2091	8.3625	13.26	<.0001
2	log10IFNa	0.1815	0.1190	-0.0519	0.4148	1.52	0.1274
3	BMI_total	-0.0467	0.0203	-0.0865	-0.0069	-2.30	0.0213
4	gender2	-0.5268	0.2227	-0.9632	-0.0904	-2.37	0.0180
5	hydroxychloroquine_ever	0.6271	0.1997	0.2357	1.0184	3.14	0.0017
6	immuno_status	1.0348	0.3579	0.3332	1.7363	2.89	0.0038
7	lung_disease	-2.9407	0.5646	-4.0473	-1.8342	-5.21	<.0001

Adjusted GEE model lnferritine with log10IFNb

Obs	Parm	Estimate	Stderr	LowerCL	UpperCL	Z	ProbZ
1	Intercept	6.5182	0.1721	6.1809	6.8555	37.88	<.0001
2	log10IFNb	0.9974	0.0805	0.8396	1.1552	12.39	<.0001
3	BMI_total	-0.0364	0.0044	-0.0451	-0.0278	-8.26	<.0001
4	arterial_hypertension	-0.3905	0.0433	-0.4752	-0.3057	-9.03	<.0001
5	diabetes	-0.4398	0.1218	-0.6784	-0.2012	-3.61	0.0003
6	gender2	-0.6906	0.2183	-1.1184	-0.2628	-3.16	0.0016
7	immuno_status	1.0568	0.2317	0.6028	1.5109	4.56	<.0001
8	lung_disease	-3.7647	0.2251	-4.2058	-3.3235	-16.72	<.0001
9	other_therapy_ever	-0.2670	0.1172	-0.4967	-0.0372	-2.28	0.0228

Adjusted GEE model lnferritine with log10IFNg

Obs	Parm	Estimate	Stderr	LowerCL	UpperCL	Z	ProbZ
1	Intercept	9.2533	0.3605	8.5468	9.9598	25.67	<.0001
2	log10IFNg	0.5510	0.1108	0.3339	0.7681	4.97	<.0001
3	Age	-0.0224	0.0027	-0.0277	-0.0170	-8.14	<.0001
4	BMI_total	-0.0577	0.0050	-0.0676	-0.0479	-11.47	<.0001
5	corticosteroids_ever	-0.1155	0.0523	-0.2180	-0.0130	-2.21	0.0271
6	diabetes	-0.3313	0.0642	-0.4572	-0.2054	-5.16	<.0001
7	gender2	-0.4672	0.2358	-0.9293	-0.0050	-1.98	0.0476
8	hydroxychloroquine_ever	0.2742	0.1005	0.0773	0.4711	2.73	0.0064
9	immuno_status	0.4902	0.2158	0.0672	0.9132	2.27	0.0231
10	lung_disease	-3.0298	0.2258	-3.4725	-2.5872	-13.42	<.0001
11	other_therapy_ever	-0.2989	0.0777	-0.4512	-0.1466	-3.85	0.0001

Adjusted GEE model Inferritine with log10IFNl23

Obs	Parm	Estimate	Stderr	LowerCL	UpperCL	Z	ProbZ
1	Intercept	9.6353	0.3576	8.9344	10.3362	26.94	<.0001
2	log10IFNl23	0.2292	0.0121	0.2055	0.2528	18.98	<.0001
3	Age	-0.0304	0.0021	-0.0345	-0.0262	-14.31	<.0001
4	BMI_total	-0.0451	0.0129	-0.0704	-0.0198	-3.50	0.0005
5	hydroxychloroquine_ever	0.4513	0.0993	0.2566	0.6459	4.54	<.0001
6	immuno_status	0.5998	0.2446	0.1204	1.0792	2.45	0.0142
7	lung_disease	-3.1794	0.1906	-3.5531	-2.8058	-16.68	<.0001
8	other_therapy_ever	-0.4826	0.2288	-0.9310	-0.0341	-2.11	0.0349

Adjusted GEE model Inferritine with log10IL10

Obs	Parm	Estimate	Stderr	LowerCL	UpperCL	Z	ProbZ
1	Intercept	8.0855	0.7801	6.5564	9.6145	10.36	<.0001
2	log10IL10	0.6809	0.3443	0.0061	1.3558	1.98	0.0480
3	Age	-0.0160	0.0038	-0.0235	-0.0085	-4.20	<.0001
4	BMI_total	-0.0315	0.0052	-0.0417	-0.0214	-6.09	<.0001
5	arterial_hypertension	-0.4274	0.1866	-0.7933	-0.0616	-2.29	0.0220
6	gender2	-0.4239	0.1239	-0.6668	-0.1811	-3.42	0.0006
7	hydroxychloroquine_ever	0.3180	0.1310	0.0612	0.5748	2.43	0.0152
8	immuno_status	0.5736	0.2127	0.1567	0.9904	2.70	0.0070
9	lung_disease	-2.6103	0.0543	-2.7168	-2.5038	-48.04	<.0001
10	other_therapy_ever	-0.3264	0.1061	-0.5342	-0.1185	-3.08	0.0021

Adjusted GEE model Inferritine with log10IL12

Obs	Parm	Estimate	Stderr	LowerCL	UpperCL	Z	ProbZ
1	Intercept	10.3480	0.5964	9.1791	11.5168	17.35	<.0001
2	log10IL12	0.2458	0.3286	-0.3984	0.8899	0.75	0.4546
3	Age	-0.0331	0.0016	-0.0362	-0.0301	-21.37	<.0001
4	BMI_total	-0.0482	0.0221	-0.0916	-0.0048	-2.18	0.0293
5	hydroxychloroquine_ever	0.4409	0.0860	0.2722	0.6095	5.12	<.0001
6	lung_disease	-2.7420	0.3458	-3.4198	-2.0643	-7.93	<.0001
7	other_therapy_ever	-0.4947	0.2250	-0.9356	-0.0537	-2.20	0.0279

Adjusted GEE model lnferritine with log10IL6

Obs	Parm	Estimate	Stderr	LowerCL	UpperCL	Z	ProbZ
1	Intercept	7.7155	1.2682	5.2298	10.2012	6.08	<.0001
2	log10IL6	0.6478	0.3239	0.0128	1.2827	2.00	0.0455
3	Age	-0.0177	0.0057	-0.0289	-0.0065	-3.10	0.0019
4	BMI_total	-0.0276	0.0093	-0.0457	-0.0094	-2.97	0.0030
5	arterial_hypertension	-0.3871	0.1717	-0.7236	-0.0506	-2.25	0.0242
6	gender2	-0.3699	0.0397	-0.4477	-0.2921	-9.32	<.0001
7	hydroxychloroquine_ever	0.3435	0.0643	0.2175	0.4694	5.34	<.0001
8	immuno_status	0.7585	0.0729	0.6155	0.9015	10.40	<.0001
9	lung_disease	-2.6099	0.0651	-2.7374	-2.4824	-40.12	<.0001
10	other_therapy_ever	-0.3289	0.1286	-0.5809	-0.0770	-2.56	0.0105

Adjusted GEE model lnferritine with log10IL8

Obs	Parm	Estimate	Stderr	LowerCL	UpperCL	Z	ProbZ
1	Intercept	9.1662	1.0181	7.1707	11.1616	9.00	<.0001
2	log10IL8	0.1454	0.2668	-0.3776	0.6683	0.54	0.5858
3	Age	-0.0237	0.0035	-0.0306	-0.0168	-6.75	<.0001
4	BMI_total	-0.0322	0.0117	-0.0551	-0.0093	-2.76	0.0058
5	arterial_hypertension	-0.3753	0.1796	-0.7273	-0.0234	-2.09	0.0366
6	diabetes	0.0504	0.0239	0.0036	0.0972	2.11	0.0349
7	gender2	-0.3531	0.0910	-0.5314	-0.1747	-3.88	0.0001
8	hydroxychloroquine_ever	0.4163	0.0400	0.3379	0.4947	10.41	<.0001
9	immuno_status	0.5781	0.2395	0.1088	1.0475	2.41	0.0158
10	lung_disease	-2.5566	0.1406	-2.8322	-2.2810	-18.18	<.0001
11	other_therapy_ever	-0.4074	0.1780	-0.7563	-0.0585	-2.29	0.0221

Adjusted GEE model Inferritine with log10IP10

Obs	Parm	Estimate	Stderr	LowerCL	UpperCL	Z	ProbZ
1	Intercept	6.9565	0.6854	5.6132	8.2998	10.15	<.0001
2	log10IP10	0.8464	0.1578	0.5371	1.1558	5.36	<.0001
3	Age	-0.0177	0.0031	-0.0237	-0.0117	-5.77	<.0001
4	BMI_total	-0.0321	0.0027	-0.0375	-0.0268	-11.79	<.0001
5	antibacterial_ever	0.1893	0.0606	0.0706	0.3081	3.12	0.0018
6	arterial_hypertension	-0.5598	0.1322	-0.8189	-0.3007	-4.23	<.0001
7	corticosteroids_ever	-0.4394	0.1066	-0.6483	-0.2305	-4.12	<.0001
8	gender2	-0.4325	0.0873	-0.6036	-0.2614	-4.95	<.0001
9	hydroxychloroquine_ever	0.3777	0.0863	0.2085	0.5469	4.38	<.0001
10	immuno_status	0.9764	0.1972	0.5899	1.3630	4.95	<.0001
11	lung_disease	-2.1199	0.2536	-2.6169	-1.6229	-8.36	<.0001
12	other_therapy_ever	-0.4923	0.1871	-0.8590	-0.1256	-2.63	0.0085

Adjusted GEE model Inferritine with log10GM

Obs	Parm	Estimate	Stderr	LowerCL	UpperCL	Z	ProbZ
1	Intercept	7.2913	0.6561	6.0053	8.5774	11.11	<.0001
2	log10GM	0.3170	0.2671	-0.2065	0.8404	1.19	0.2353
3	BMI_total	-0.0484	0.0195	-0.0866	-0.0102	-2.48	0.0130
4	gender2	-0.5331	0.1829	-0.8915	-0.1747	-2.92	0.0036
5	hydroxychloroquine_ever	0.5791	0.1848	0.2169	0.9412	3.13	0.0017
6	immuno_status	1.0409	0.3166	0.4204	1.6615	3.29	0.0010
7	lung_disease	-3.1729	0.4590	-4.0725	-2.2732	-6.91	<.0001
