# Supplementary material for: Comprehensive antibody and cytokine profiling in hospitalized COVID-19 patients in relation to clinical outcomes in a large Belgian cohort
Source: Sci Rep. 2023 Nov 7;13:19322. doi: 10.1038/s41598-023-46421-4 (PMC10630327; doi:10.1038/s41598-023-46421-4)
Supplement: Supplementary file 1 — Supplementary Information. [file 41598_2023_46421_MOESM1_ESM.zip › Adjusted GEE model for Ln(PF-ratio) with AB.pdf]

Adjusted GEE model for lnpf_ratio with IgG_sero

Obs	Parm	Estimate	Stderr	LowerCL	UpperCL	Z	ProbZ
1	Intercept	6.2025	0.0720	6.0615	6.3436	86.17	<.0001
2	IgG_sero	-0.0219	0.0824	-0.1833	0.1396	-0.27	0.7905
3	BMI_total	-0.0088	0.0031	-0.0150	-0.0026	-2.80	0.0051
4	antibacterial_ever	-0.2374	0.0428	-0.3213	-0.1535	-5.54	<.0001
5	diabetes	0.0898	0.0321	0.0268	0.1527	2.80	0.0052
6	gender2	0.1664	0.0252	0.1170	0.2157	6.61	<.0001
7	hydroxychloroquine_ever	-0.1854	0.0487	-0.2808	-0.0900	-3.81	0.0001
8	immuno_status	-0.2721	0.0843	-0.4374	-0.1069	-3.23	0.0013
9	lung_disease	-0.1951	0.0695	-0.3314	-0.0588	-2.81	0.0050
10	other_therapy_ever	-0.6484	0.1267	-0.8967	-0.4001	-5.12	<.0001

Adjusted GEE model for lnpf_ratio with IgM_sero

Obs	Parm	Estimate	Stderr	LowerCL	UpperCL	Z	ProbZ
1	Intercept	6.1220	0.1033	5.9196	6.3244	59.29	<.0001
2	IgM_sero	0.0238	0.0953	-0.1630	0.2106	0.25	0.8027
3	BMI_total	-0.0080	0.0036	-0.0151	-0.0009	-2.21	0.0269
4	antibacterial_ever	-0.2351	0.0630	-0.3585	-0.1117	-3.73	0.0002
5	corticosteroids_ever	-0.2370	0.1109	-0.4544	-0.0196	-2.14	0.0326
6	diabetes	0.1196	0.0361	0.0488	0.1903	3.31	0.0009
7	gender2	0.1055	0.0327	0.0414	0.1696	3.23	0.0013
8	kidney_injury	0.1959	0.0498	0.0984	0.2934	3.94	<.0001
9	lung_disease	-0.2012	0.0697	-0.3377	-0.0646	-2.89	0.0039
10	other_therapy_ever	-0.6334	0.1414	-0.9106	-0.3562	-4.48	<.0001

Adjusted GEE model for lnpf_ratio with log10IgG

Obs	Parm	Estimate	Stderr	LowerCL	UpperCL	Z	ProbZ
1	Intercept	6.1598	0.0443	6.0729	6.2467	138.93	<.0001
2	IgG_NIBSC_avg	0.0526	0.0569	-0.0589	0.1641	0.92	0.3550
3	BMI_total	-0.0078	0.0020	-0.0118	-0.0039	-3.86	0.0001
4	antibacterial_ever	-0.2822	0.0438	-0.3680	-0.1964	-6.45	<.0001
5	diabetes	0.0867	0.0367	0.0147	0.1587	2.36	0.0182
6	gender2	0.1448	0.0354	0.0755	0.2141	4.10	<.0001
7	hydroxychloroquine_ever	-0.1881	0.0505	-0.2871	-0.0892	-3.73	0.0002
8	immuno_status	-0.2422	0.1085	-0.4549	-0.0294	-2.23	0.0257
9	kidney_injury	0.0885	0.0437	0.0027	0.1742	2.02	0.0432
10	lung_disease	-0.1917	0.0715	-0.3319	-0.0515	-2.68	0.0074
11	other_therapy_ever	-0.6449	0.1384	-0.9161	-0.3736	-4.66	<.0001

Adjusted GEE model for lnpf_ratio with log10IgM

Obs	Parm	Estimate	Stderr	LowerCL	UpperCL	Z	ProbZ
1	Intercept	6.1134	0.0818	5.9531	6.2737	74.76	<.0001
2	IgM_NIBSC_avg	0.0293	0.0256	-0.0208	0.0794	1.15	0.2520
3	BMI_total	-0.0073	0.0034	-0.0139	-0.0007	-2.18	0.0293
4	antibacterial_ever	-0.3134	0.0359	-0.3838	-0.2431	-8.73	<.0001
5	diabetes	0.1287	0.0401	0.0502	0.2073	3.21	0.0013
6	gender2	0.0989	0.0413	0.0180	0.1799	2.40	0.0165
7	kidney_injury	0.1506	0.0499	0.0529	0.2484	3.02	0.0025
8	lung_disease	-0.2164	0.0514	-0.3172	-0.1156	-4.21	<.0001
9	other_therapy_ever	-0.6915	0.1363	-0.9586	-0.4245	-5.08	<.0001
