# Supplementary material for: Comprehensive antibody and cytokine profiling in hospitalized COVID-19 patients in relation to clinical outcomes in a large Belgian cohort
Source: Sci Rep. 2023 Nov 7;13:19322. doi: 10.1038/s41598-023-46421-4 (PMC10630327; doi:10.1038/s41598-023-46421-4)
Supplement: Supplementary file 1 — Supplementary Information. [file 41598_2023_46421_MOESM1_ESM.zip › Adjusted GEE model for Ln(PF-ratio) with CYT.pdf]

# Adjusted GEE model lnpf_ratio with log10IFNl1

| Obs | Parm | Estimate | Stderr | LowerCL | UpperCL | Z | ProbZ |
|---|---|---|---|---|---|---|---|
| 1 | Intercept | 6.4617 | 0.4043 | 5.6694 | 7.2541 | 15.98 | <.0001 |
| 2 | log10IFNL1 | -0.3711 | 0.2541 | -0.8692 | 0.1269 | -1.46 | 0.1441 |
| 3 | antibacterial_ever | -0.4261 | 0.0910 | -0.6045 | -0.2478 | -4.68 | <.0001 |
| 4 | diabetes | 0.3827 | 0.1119 | 0.1634 | 0.6019 | 3.42 | 0.0006 |
| 5 | kidney_injury | 0.3390 | 0.1335 | 0.0774 | 0.6007 | 2.54 | 0.0111 |
| 6 | malignancies | 0.3728 | 0.0346 | 0.3051 | 0.4406 | 10.78 | <.0001 |
| 7 | other_therapy_ever | -0.4917 | 0.1377 | -0.7615 | -0.2218 | -3.57 | 0.0004 |

# Adjusted GEE model lnpf_ratio with log10IFNa

| Obs | Parm | Estimate | Stderr | LowerCL | UpperCL | Z | ProbZ |
|---|---|---|---|---|---|---|---|
| 1 | Intercept | 5.4064 | 0.1158 | 5.1795 | 5.6333 | 46.70 | <.0001 |
| 2 | log10IFNa | 0.2025 | 0.0913 | 0.0236 | 0.3815 | 2.22 | 0.0265 |
| 3 | BMI_total | 0.0041 | 0.0019 | 0.0004 | 0.0078 | 2.15 | 0.0315 |
| 4 | antibacterial_ever | -0.2405 | 0.0583 | -0.3548 | -0.1262 | -4.12 | <.0001 |
| 5 | diabetes | 0.1967 | 0.0691 | 0.0612 | 0.3322 | 2.85 | 0.0044 |
| 6 | kidney_injury | 0.1776 | 0.0847 | 0.0117 | 0.3435 | 2.10 | 0.0359 |
| 7 | lung_disease | -0.6117 | 0.0759 | -0.7605 | -0.4629 | -8.06 | <.0001 |
| 8 | malignancies | 0.1848 | 0.0452 | 0.0963 | 0.2733 | 4.09 | <.0001 |
| 9 | other_therapy_ever | -0.5319 | 0.1158 | -0.7590 | -0.3049 | -4.59 | <.0001 |

## Adjusted GEE model lnpf_ratio with log10IFNb



| Obs | Parm | Estimate | Stderr | LowerCL | UpperCL | Z | ProbZ |
|---|---|---|---|---|---|---|---|
| 1 | Intercept | 6.2178 | 0.2346 | 5.7580 | 6.6776 | 26.50 | <.0001 |
| 2 | log10IFNb | -0.2183 | 0.1492 | -0.5108 | 0.0741 | -1.46 | 0.1434 |
| 3 | antibacterial_ever | -0.4872 | 0.0929 | -0.6692 | -0.3052 | -5.25 | <.0001 |
| 4 | diabetes | 0.3434 | 0.1458 | 0.0577 | 0.6292 | 2.36 | 0.0185 |
| 5 | kidney_injury | 0.3018 | 0.1366 | 0.0341 | 0.5694 | 2.21 | 0.0271 |
| 6 | malignancies | 0.3675 | 0.0405 | 0.2882 | 0.4468 | 9.08 | <.0001 |
| 7 | other_therapy_ever | -0.4862 | 0.1486 | -0.7775 | -0.1950 | -3.27 | 0.0011 |

# Adjusted GEE model lnpf_ratio with log10IFNg



| Obs | Parm | Estimate | Stderr | LowerCL | UpperCL | Z | ProbZ |
|---|---|---|---|---|---|---|---|
| 1 | Intercept | 5.9629 | 0.1542 | 5.6608 | 6.2651 | 38.68 | <.0001 |
| 2 | log10IFNg | -0.0783 | 0.1101 | -0.2941 | 0.1374 | -0.71 | 0.4768 |
| 3 | antibacterial_ever | -0.4946 | 0.0808 | -0.6530 | -0.3362 | -6.12 | <.0001 |
| 4 | diabetes | 0.4277 | 0.0919 | 0.2477 | 0.6077 | 4.66 | <.0001 |
| 5 | kidney_injury | 0.2948 | 0.1267 | 0.0466 | 0.5431 | 2.33 | 0.0199 |
| 6 | malignancies | 0.3593 | 0.0443 | 0.2725 | 0.4462 | 8.11 | <.0001 |
| 7 | other_therapy_ever | -0.5168 | 0.1207 | -0.7533 | -0.2803 | -4.28 | <.0001 |

# Adjusted GEE model lnpf_ratio with log10IFNl23



| Obs | Parm | Estimate | Stderr | LowerCL | UpperCL | Z | ProbZ |
|---|---|---|---|---|---|---|---|
| 1 | Intercept | 6.3528 | 0.5038 | 5.3653 | 7.3402 | 12.61 | <.0001 |
| 2 | log10IFNl23 | -0.2558 | 0.2580 | -0.7614 | 0.2498 | -0.99 | 0.3213 |
| 3 | antibacterial_ever | -0.5446 | 0.0887 | -0.7184 | -0.3707 | -6.14 | <.0001 |
| 4 | diabetes | 0.4674 | 0.1062 | 0.2592 | 0.6756 | 4.40 | <.0001 |
| 5 | kidney_injury | 0.2702 | 0.1367 | 0.0022 | 0.5382 | 1.98 | 0.0482 |
| 6 | malignancies | 0.3762 | 0.0491 | 0.2800 | 0.4724 | 7.67 | <.0001 |
| 7 | other_therapy_ever | -0.5584 | 0.1092 | -0.7724 | -0.3444 | -5.11 | <.0001 |

## Adjusted GEE model lnpf_ratio with log10IL10



| Obs | Parm | Estimate | Stderr | LowerCL | UpperCL | Z | ProbZ |
|---|---|---|---|---|---|---|---|
| 1 | Intercept | 6.3243 | 0.1089 | 6.1108 | 6.5377 | 58.07 | <.0001 |
| 2 | log10IL10 | -0.4250 | 0.1097 | -0.6400 | -0.2100 | -3.87 | 0.0001 |
| 3 | antibacterial_ever | -0.3417 | 0.0572 | -0.4538 | -0.2295 | -5.97 | <.0001 |
| 4 | diabetes | 0.4484 | 0.1000 | 0.2524 | 0.6444 | 4.48 | <.0001 |
| 5 | kidney_injury | 0.2800 | 0.0945 | 0.0948 | 0.4652 | 2.96 | 0.0030 |
| 6 | malignancies | 0.3816 | 0.0314 | 0.3200 | 0.4433 | 12.14 | <.0001 |
| 7 | other_therapy_ever | -0.5078 | 0.1134 | -0.7301 | -0.2855 | -4.48 | <.0001 |

## Adjusted GEE model lnpf_ratio with log10IL12



| Obs | Parm | Estimate | Stderr | LowerCL | UpperCL | Z | ProbZ |
|---|---|---|---|---|---|---|---|
| 1 | Intercept | 5.7363 | 0.0440 | 5.6500 | 5.8226 | 130.30 | <.0001 |
| 2 | log10IL12 | 0.2236 | 0.0862 | 0.0547 | 0.3925 | 2.60 | 0.0095 |
| 3 | antibacterial_ever | -0.4994 | 0.0758 | -0.6480 | -0.3508 | -6.59 | <.0001 |
| 4 | diabetes | 0.3999 | 0.1034 | 0.1972 | 0.6026 | 3.87 | 0.0001 |
| 5 | kidney_injury | 0.2643 | 0.1223 | 0.0245 | 0.5041 | 2.16 | 0.0307 |
| 6 | malignancies | 0.4086 | 0.0356 | 0.3389 | 0.4783 | 11.49 | <.0001 |
| 7 | other_therapy_ever | -0.4944 | 0.0903 | -0.6713 | -0.3175 | -5.48 | <.0001 |

# Adjusted GEE model lnpf_ratio with log10IL6

| Obs | Parm | Estimate | Stderr | LowerCL | UpperCL | Z | ProbZ |
|---|---|---|---|---|---|---|---|
| 1 | Intercept | 6.0047 | 0.1218 | 5.7660 | 6.2435 | 49.29 | <.0001 |
| 2 | log10IL6 | -0.2956 | 0.0435 | -0.3809 | -0.2104 | -6.80 | <.0001 |
| 3 | BMI_total | 0.0072 | 0.0032 | 0.0009 | 0.0135 | 2.24 | 0.0249 |
| 4 | antibacterial_ever | -0.1416 | 0.0591 | -0.2574 | -0.0258 | -2.40 | 0.0166 |
| 5 | diabetes | 0.3071 | 0.0725 | 0.1650 | 0.4492 | 4.24 | <.0001 |
| 6 | kidney_injury | 0.3216 | 0.0589 | 0.2061 | 0.4371 | 5.46 | <.0001 |
| 7 | lung_disease | -0.5435 | 0.2088 | -0.9527 | -0.1344 | -2.60 | 0.0092 |
| 8 | malignancies | 0.2638 | 0.0317 | 0.2016 | 0.3260 | 8.31 | <.0001 |
| 9 | other_therapy_ever | -0.5449 | 0.1025 | -0.7458 | -0.3439 | -5.31 | <.0001 |

# Adjusted GEE model lnpf_ratio  with log10IL8

| Obs | Parm | Estimate | Stderr | LowerCL | UpperCL | Z | ProbZ |
|---|---|---|---|---|---|---|---|
| 1 | Intercept | 6.5493 | 0.1384 | 6.2781 | 6.8205 | 47.33 | <.0001 |
| 2 | log10IL8 | -0.4040 | 0.0937 | -0.5876 | -0.2203 | -4.31 | <.0001 |
| 3 | antibacterial_ever | -0.3823 | 0.0599 | -0.4997 | -0.2648 | -6.38 | <.0001 |
| 4 | diabetes | 0.4421 | 0.0880 | 0.2696 | 0.6146 | 5.02 | <.0001 |
| 5 | kidney_injury | 0.3383 | 0.1021 | 0.1383 | 0.5384 | 3.32 | 0.0009 |
| 6 | malignancies | 0.3992 | 0.0405 | 0.3197 | 0.4787 | 9.84 | <.0001 |
| 7 | other_therapy_ever | -0.4974 | 0.1058 | -0.7047 | -0.2900 | -4.70 | <.0001 |

## Adjusted GEE model lnpf_ratio with log10IP10



| Obs | Parm | Estimate | Stderr | LowerCL | UpperCL | Z | ProbZ |
|---|---|---|---|---|---|---|---|
| 1 | Intercept | 6.9373 | 0.2602 | 6.4272 | 7.4473 | 26.66 | <.0001 |
| 2 | log10IP10 | -0.4173 | 0.1174 | -0.6474 | -0.1871 | -3.55 | 0.0004 |
| 3 | antibacterial_ever | -0.3487 | 0.1420 | -0.6269 | -0.0704 | -2.46 | 0.0140 |
| 4 | diabetes | 0.2714 | 0.0970 | 0.0813 | 0.4616 | 2.80 | 0.0051 |
| 5 | malignancies | 0.2869 | 0.0542 | 0.1807 | 0.3931 | 5.29 | <.0001 |
| 6 | other_therapy_ever | -0.4290 | 0.1550 | -0.7327 | -0.1253 | -2.77 | 0.0056 |

## Adjusted GEE model lnpf_ratio with log10GM



| Obs | Parm | Estimate | Stderr | LowerCL | UpperCL | Z | ProbZ |
|---|---|---|---|---|---|---|---|
| 1 | Intercept | 5.6557 | 0.2476 | 5.1704 | 6.1409 | 22.85 | <.0001 |
| 2 | log10GM | 0.1833 | 0.2370 | -0.2811 | 0.6478 | 0.77 | 0.4391 |
| 3 | antibacterial_ever | -0.3996 | 0.0910 | -0.5781 | -0.2212 | -4.39 | <.0001 |
| 4 | corticosteroids_ever | -0.3713 | 0.1530 | -0.6712 | -0.0714 | -2.43 | 0.0152 |
| 5 | diabetes | 0.3707 | 0.1377 | 0.1008 | 0.6407 | 2.69 | 0.0071 |
| 6 | kidney_injury | 0.3324 | 0.1109 | 0.1150 | 0.5497 | 3.00 | 0.0027 |
| 7 | malignancies | 0.4299 | 0.1211 | 0.1926 | 0.6673 | 3.55 | 0.0004 |
| 8 | other_therapy_ever | -0.5019 | 0.1584 | -0.8124 | -0.1915 | -3.17 | 0.0015 |
