# Supplementary material for: Comprehensive antibody and cytokine profiling in hospitalized COVID-19 patients in relation to clinical outcomes in a large Belgian cohort
Source: Sci Rep. 2023 Nov 7;13:19322. doi: 10.1038/s41598-023-46421-4 (PMC10630327; doi:10.1038/s41598-023-46421-4)
Supplement: Supplementary file 1 — Supplementary Information. [file 41598_2023_46421_MOESM1_ESM.zip › Adjusted GEE model for Ln(White Blood Cells) with AB.pdf]

Adjusted GEE model for lnwhite_BC with IgG_sero

Obs	Parm	Estimate	Stderr	LowerCL	UpperCL	Z	ProbZ
1	Intercept	1.8028	0.0403	1.7237	1.8818	44.69	<.0001
2	IgG_sero	0.1950	0.0529	0.0913	0.2987	3.69	0.0002
3	corticosteroids_ever	0.1154	0.0514	0.0146	0.2162	2.24	0.0248

Adjusted GEE model for lnwhite_BC with IgM_sero

Obs	Parm	Estimate	Stderr	LowerCL	UpperCL	Z	ProbZ
1	Intercept	1.7705	0.0432	1.6858	1.8553	40.94	<.0001
2	IgM_sero	0.1795	0.0260	0.1285	0.2305	6.89	<.0001
3	corticosteroids_ever	0.1249	0.0523	0.0223	0.2275	2.39	0.0170
4	hydroxychloroquine_ever	-0.0544	0.0201	-0.0939	-0.0150	-2.70	0.0069

Adjusted GEE model for lnwhite_BC with log10IgG

Obs	Parm	Estimate	Stderr	LowerCL	UpperCL	Z	ProbZ
1	Intercept	1.7736	0.0503	1.6749	1.8723	35.23	<.0001
2	IgG_NIBSC_avg	0.1123	0.0290	0.0554	0.1691	3.87	0.0001
3	corticosteroids_ever	0.1212	0.0515	0.0203	0.2221	2.35	0.0186

Adjusted GEE model for lnwhite_BC with log10IgM

Obs	Parm	Estimate	Stderr	LowerCL	UpperCL	Z	ProbZ
1	Intercept	1.7566	0.0618	1.6356	1.8777	28.45	<.0001
2	IgM_NIBSC_avg	0.0678	0.0172	0.0341	0.1015	3.94	<.0001
3	corticosteroids_ever	0.1295	0.0558	0.0200	0.2389	2.32	0.0204
4	hydroxychloroquine_ever	-0.0508	0.0241	-0.0981	-0.0034	-2.10	0.0355
