# Supplementary material for: Comprehensive antibody and cytokine profiling in hospitalized COVID-19 patients in relation to clinical outcomes in a large Belgian cohort
Source: Sci Rep. 2023 Nov 7;13:19322. doi: 10.1038/s41598-023-46421-4 (PMC10630327; doi:10.1038/s41598-023-46421-4)
Supplement: Supplementary file 1 — Supplementary Information. [file 41598_2023_46421_MOESM1_ESM.zip › Adjusted GEE model for Ln(White Blood Cells) with CYT.pdf]

Adjusted GEE model lnwhite_BC with log10IFNl1

Obs	Parm	Estimate	Stderr	LowerCL	UpperCL	Z	ProbZ
1	Intercept	1.7654	0.1180	1.5340	1.9967	14.95	<.0001
2	log10IFNL1	0.0739	0.0703	-0.0639	0.2117	1.05	0.2930
3	antibacterial_ever	0.1352	0.0269	0.0824	0.1880	5.02	<.0001
4	hydroxychloroquine_ever	-0.1370	0.0384	-0.2122	-0.0617	-3.57	0.0004
5	kidney_injury	-0.1336	0.0400	-0.2119	-0.0553	-3.34	0.0008
6	lung_disease	0.1591	0.0684	0.0251	0.2931	2.33	0.0200

Adjusted GEE model lnwhite_BC with log10IFNa

Obs	Parm	Estimate	Stderr	LowerCL	UpperCL	Z	ProbZ
1	Intercept	2.0460	0.0248	1.9974	2.0945	82.66	<.0001
2	log10IFNa	-0.1818	0.0343	-0.2491	-0.1145	-5.29	<.0001
3	antibacterial_ever	0.1583	0.0195	0.1200	0.1965	8.11	<.0001
4	hydroxychloroquine_ever	-0.1394	0.0451	-0.2278	-0.0509	-3.09	0.0020

Adjusted GEE model lnwhite_BC with log10IFNb

Obs	Parm	Estimate	Stderr	LowerCL	UpperCL	Z	ProbZ
1	Intercept	1.7714	0.0563	1.6611	1.8818	31.46	<.0001
2	log10IFNb	0.0832	0.0229	0.0384	0.1281	3.64	0.0003
3	antibacterial_ever	0.1405	0.0209	0.0995	0.1815	6.72	<.0001
4	hydroxychloroquine_ever	-0.1518	0.0458	-0.2415	-0.0621	-3.32	0.0009
5	kidney_injury	-0.1660	0.0307	-0.2263	-0.1058	-5.40	<.0001

Adjusted GEE model lnwhite_BC with log10IFNg

Obs	Parm	Estimate	Stderr	LowerCL	UpperCL	Z	ProbZ
1	Intercept	2.4316	0.0549	2.3239	2.5393	44.25	<.0001
2	log10IFNg	-0.1294	0.0476	-0.2226	-0.0362	-2.72	0.0065
3	BMI_total	-0.0104	0.0039	-0.0181	-0.0027	-2.64	0.0082
4	antibacterial_ever	0.0968	0.0119	0.0735	0.1200	8.15	<.0001
5	kidney_injury	-0.1020	0.0502	-0.2004	-0.0035	-2.03	0.0424
6	lung_disease	0.2710	0.0538	0.1655	0.3765	5.03	<.0001
7	other_therapy_ever	-0.2107	0.0516	-0.3118	-0.1096	-4.08	<.0001

Adjusted GEE model lnwhite_BC with log10IFNl23

Obs	Parm	Estimate	Stderr	LowerCL	UpperCL	Z	ProbZ
1	Intercept	2.4123	0.1062	2.2040	2.6205	22.71	<.0001
2	log10IFNl23	-0.0811	0.0562	-0.1912	0.0290	-1.44	0.1489
3	BMI_total	-0.0118	0.0036	-0.0189	-0.0047	-3.26	0.0011
4	antibacterial_ever	0.1166	0.0216	0.0742	0.1590	5.40	<.0001
5	hydroxychloroquine_ever	-0.0886	0.0445	-0.1758	-0.0015	-1.99	0.0462
6	immuno_status	-0.0596	0.0252	-0.1091	-0.0102	-2.36	0.0180
7	kidney_injury	-0.1288	0.0593	-0.2450	-0.0125	-2.17	0.0299
8	lung_disease	0.2425	0.0787	0.0883	0.3966	3.08	0.0021
9	other_therapy_ever	-0.1566	0.0777	-0.3089	-0.0044	-2.02	0.0438

Adjusted GEE model lnwhite_BC with log10IL10

Obs	Parm	Estimate	Stderr	LowerCL	UpperCL	Z	ProbZ
1	Intercept	1.7833	0.0541	1.6772	1.8893	32.95	<.0001
2	log10IL10	0.0996	0.0402	0.0209	0.1783	2.48	0.0132
3	antibacterial_ever	0.1092	0.0177	0.0746	0.1438	6.19	<.0001
4	hydroxychloroquine_ever	-0.1400	0.0401	-0.2186	-0.0614	-3.49	0.0005
5	kidney_injury	-0.1418	0.0425	-0.2252	-0.0584	-3.33	0.0009
6	lung_disease	0.1571	0.0608	0.0380	0.2762	2.59	0.0097

Adjusted GEE model lnwhite_BC with log10IL12

Obs	Parm	Estimate	Stderr	LowerCL	UpperCL	Z	ProbZ
1	Intercept	1.8745	0.0296	1.8164	1.9325	63.29	<.0001
2	log10IL12	0.0561	0.0647	-0.0707	0.1830	0.87	0.3858
3	antibacterial_ever	0.1292	0.0192	0.0915	0.1668	6.72	<.0001
4	hydroxychloroquine_ever	-0.1313	0.0384	-0.2065	-0.0561	-3.42	0.0006
5	kidney_injury	-0.1359	0.0402	-0.2148	-0.0570	-3.38	0.0007
6	lung_disease	0.1706	0.0788	0.0162	0.3250	2.17	0.0303

Adjusted GEE model lnwhite_BC with log10IL6

Obs	Parm	Estimate	Stderr	LowerCL	UpperCL	Z	ProbZ
1	Intercept	1.5818	0.0292	1.5245	1.6391	54.09	<.0001
2	log10IL6	0.2300	0.0222	0.1865	0.2736	10.35	<.0001
3	hydroxychloroquine_ever	-0.1413	0.0311	-0.2022	-0.0804	-4.55	<.0001
4	kidney_injury	-0.1697	0.0393	-0.2467	-0.0926	-4.32	<.0001
5	lung_disease	0.1481	0.0462	0.0576	0.2386	3.21	0.0013

Adjusted GEE model lnwhite_BC with log10IL8

Obs	Parm	Estimate	Stderr	LowerCL	UpperCL	Z	ProbZ
1	Intercept	1.8596	0.0696	1.7233	1.9960	26.74	<.0001
2	log10IL8	0.0148	0.0563	-0.0955	0.1252	0.26	0.7925
3	antibacterial_ever	0.1292	0.0296	0.0713	0.1872	4.37	<.0001
4	hydroxychloroquine_ever	-0.1318	0.0316	-0.1937	-0.0698	-4.17	<.0001
5	kidney_injury	-0.1367	0.0394	-0.2139	-0.0594	-3.47	0.0005
6	lung_disease	0.1631	0.0639	0.0379	0.2882	2.55	0.0107

Adjusted GEE model lnwhite_BC with log10IP10

Obs	Parm	Estimate	Stderr	LowerCL	UpperCL	Z	ProbZ
1	Intercept	2.6850	0.1010	2.4872	2.8829	26.60	<.0001
2	log10IP10	-0.1755	0.0465	-0.2667	-0.0843	-3.77	0.0002
3	BMI_total	-0.0117	0.0036	-0.0188	-0.0047	-3.25	0.0011
4	antibacterial_ever	0.1332	0.0263	0.0817	0.1847	5.07	<.0001
5	diabetes	-0.0849	0.0234	-0.1308	-0.0389	-3.62	0.0003
6	immuno_status	-0.0857	0.0140	-0.1132	-0.0582	-6.11	<.0001
7	lung_disease	0.2772	0.0521	0.1750	0.3794	5.32	<.0001
8	other_therapy_ever	-0.1665	0.0682	-0.3002	-0.0328	-2.44	0.0146

Adjusted GEE model lnwhite_BC with log10GM

Obs	Parm	Estimate	Stderr	LowerCL	UpperCL	Z	ProbZ
1	Intercept	1.9497	0.0464	1.8588	2.0406	42.06	<.0001
2	log10GM	-0.0728	0.0349	-0.1412	-0.0044	-2.09	0.0371
3	antibacterial_ever	0.1301	0.0212	0.0884	0.1717	6.12	<.0001
4	hydroxychloroquine_ever	-0.1278	0.0350	-0.1964	-0.0591	-3.65	0.0003
5	kidney_injury	-0.1385	0.0428	-0.2223	-0.0547	-3.24	0.0012
6	lung_disease	0.1709	0.0727	0.0283	0.3134	2.35	0.0188
