# Supplementary material for: Comprehensive antibody and cytokine profiling in hospitalized COVID-19 patients in relation to clinical outcomes in a large Belgian cohort
Source: Sci Rep. 2023 Nov 7;13:19322. doi: 10.1038/s41598-023-46421-4 (PMC10630327; doi:10.1038/s41598-023-46421-4)
Supplement: Supplementary file 1 — Supplementary Information. [file 41598_2023_46421_MOESM1_ESM.zip › supplementary read me instruction file.docx]

# Comprehensive antibody and cytokine profiling in COVID-19 patients in relation to clinical outcomes in a large Belgian

Guide for reading the pdf documents related to the adjusted GEE models

This document serves a guide for reading the pdf documents related to the results from the statistical models. The statistical models that were used are called generalized estimating equations (GEE). This manuscript reports the effect of 2 antibodies (Abs) and 11 cytokines (CYTs) on 13 outcomes. For the statistical analysis, continuous outcome measures were transformed using a natural logarithm (ln), while CYTs and ABs levels were log_10_ transformed. As mentioned in the main text, the GEE models including either a CYT or AB are adjusted for patient characteristics, comorbidities and therapies through a backward model selection procedure. As a consequence, besides the CYT or AB that remains in the model (even if it does not statistically significant influence the outcome measure), only statistically significant patient characteristics, comorbidities or therapies will remain in the model. For more information on the statistical method, we refer you to the main text of the manuscript.

Because of the extensive statistical analyses conducted, we structured the documents as follows:

- Statistical models for 1 outcome are reported in a pdf document, separately for CYTs and ABs. For instance, the statistical models in which the effect of cytokines on the hospital mortality are investigated are found in the pdf document called ‘Adjusted GEE model for *hospital mortality* with *CYT*’. Similarly for antibodies, the corresponding pdf document is called ‘Adjusted GEE model for *hospital mortality* with *AB’.*  In total there are 26 documents for 13 outcomes reporting the results from the adjusted GEE models, separately for antibodies and cytokines.
- A given pdf document, let’s say ‘Adjusted GEE model for *hospital mortality* with *CYT*’, reports per page the Adjusted GEE model per cytokine. The title above each table with results, indicates clearly which cytokine is included in the statistical model.
- The results for each adjusted GEE model are reported in a table which contains the following columns:
  - Obs: indicates the row number
  - Parm: indicates the name of the covariate. In the glossary below, more information is found on the naming of the covariates. As mentioned previously, the respective CYT or AB are log_10_-transformed. This is indicated with the prefix ‘log10’ to the name of the CYT or AB. If not indicated specifically, the CYT or AB were still log_10_-transformed.
  - Estimate: This is the parameter estimate for a given covariate in the statistical model.
  - Stderr: this is the standard error for the corresponding estimate
  - LowerCL: is the lower limit of the 95% confidence interval.
  - UpperCL: is the upper limit of the 95% confidence interval.
  - Z: z-score.
  - ProbZ: indicates the p-value for a parameter estimate.

The glossary below gives a more detailed explanation for the naming of the covariates found in the statistical model:

| **Reported names** | **Explanation** |
| --- | --- |
| **Outcomes** | |
| Mortality_30d | 30-day mortality |
| Mortality_90d | 90-day mortality |
| Hospital mortality | Hospital mortality |
| ICU_admission | Admitted to intensive care unit |
| ICU_mortality | Mortality intensive care unit |
| Lncrp | Natural logarithm of CRP levels |
| Lnpf_ratio | Natural logarithm of P/F-ratio |
| Lnwhite_BC | Natural logarithm of white blood cell levels |
| Lnd_dimers | Natural logarithm of D-dimer levels |
| lnferritin | Natural logarithm of Ferritin levels |
| **Antibodies** | |
| IgG_sero | Seropositivity for IgG |
| IgM_Sero | Seropositivity for IgM |
| IgG_NIBSC_avg | Log_10_-transformed IgG levels |
| IgM_NIBSC_avg | Log_10_-transformed IgM levels |
| **Cytokines** | |
| Log10IFNl1 | Log_10_-transformed IFN-λ1 levels |
| Log10IFNa | Log_10_-transformed IFN-α2 levels |
| Log10IFNb | Log_10_-transformed IFN-β levels |
| Log10IFNg | Log_10_-transformed IFN-γ levels |
| Log10IFNl23 | Log_10_-transformed IFN-λ2/3 levels |
| Log10IL10 | Log_10_-transformed IL-10 levels |
| Log10IL12 | Log_10_-transformed IL-12p70 levels |
| Log10IL6 | Log_10_-transformed IL-6 levels |
| Log10IL8 | Log_10_-transformed IL-8 levels |
| Log10IP10 | Log_10_-transformed IP-10 levels |
| Log10GM | Log_10_-transformed GM-CSF levels |
| **Patient Characteristics, comorbidities, and therapies** | |
| Age | Age of the patient |
| Gender2 | 0= Male, 1= Female |
| BMI_total | Body Mass Index (BMI) |
| Diabetes | Is diabetes treated with medication present? (0= No, 1= Yes) |
| Lung_disease | Is COPD or interstitial lung disease treated by medication for more than 6 months present? (0= No, 1= Yes) |
| Kidney_injury | Is Chronic kidney injury present? (0= No, 1= Yes) |
| Arterial_hypertension | Is arterial hypertension treated with medication present? (0= No, 1= Yes) |
| Immuno_status | Is an immunocompromised status present (long term immunosuppressive medication, steroids, chemotherapy/radiotherapy)? (0= No, 1= Yes) |
| malignancies | Were malignancies present? (0= No, 1= Yes) |
| Antibacterial_ever | Was antibacterial therapy ever taken? (0= No, 1= Yes) |
| hydroxychloroquine_ever | Were hydroxychloroquines ever taken? (0= No, 1= Yes) |
| corticosteroids_ever | Were corticosteroids ever taken? (0= No, 1= Yes) |
| Other_therapy_ever | Were other therapies used then ? (0= No, 1= Yes) |
